# Minimal impact of recent decline in C_4_ vegetation abundance on atmospheric carbon isotopic composition

**DOI:** 10.1038/s43247-025-03102-6

**Published:** 2026-01-10

**Authors:** Aliénor Lavergne, Sandy P. Harrison, Kamolphat Atsawawaranunt, Ning Dong, Iain Colin Prentice

**Affiliations:** 1 Department of Geography and Environmental Science, University of Reading, Berkshire, UK; 2 Ministry of Education Key Laboratory for Earth System Modeling, Department of Earth System Science, Tsinghua University, Beijing, China; 3 Department of Biological Sciences, City Campus, Auckland University, Auckland, New Zealand; 4 College of Resources and Environment, Huazhong Agricultural University, Wuhan, 430070, China; 5 Georgina Mace Centre for the Living Planet, Department of Life Sciences, Imperial College London, Silwood Park Campus, Ascot, UK; 6 Present Address: Nature Geoscience, Springer Nature Group, The Campus, London, UK

**Keywords:** Biogeography, Climate-change ecology, Ecological modelling, Stable isotope analysis

## Abstract

Changes in atmospheric carbon dioxide concentrations, climate, and land management influence the abundance and distribution of C_3_ and C_4_ plants, yet their impact on the global carbon cycle remains uncertain. Here, we use a parsimonious model of C_3_ and C_4_ plant distribution, based on optimality principles, combined with a simplified representation of the global carbon cycle, to assess how shifts in plant abundances driven by carbon dioxide and climate affect global gross primary production, land carbon isotope discrimination, and the isotopic composition of atmospheric carbon dioxide. We estimate that the proportion of C_4_ plants in total biomass declined from about 16% to 12% between 1982 and 2016, despite an increase in the abundance of C_4_ crops. This decline reflects the reduced competitive advantage of C_4_ photosynthesis in a carbon dioxide-enriched atmosphere. As a result, global gross primary production rose by approximately 16.5 ± 1.8 petagrams of carbon, and land carbon isotope discrimination increased by 0.017 ± 0.001‰ per year. Accounting for changes in C_3_ and C_4_ abundances reduces the difference between observed and modeled trends in atmospheric carbon isotope composition, but does not fully explain the observed decrease, pointing to additional, unaccounted drivers.

## Abstract (web summary)

The fraction of C4 plants in terrestrial biomass decreased from 16% to 12% between 1982 and 2016, but the change is too small to explain the observed change in the isotopic composition of atmospheric carbon dioxide, according to estimates based on a C3/C4 distribution model and a carbon cycle box model.

## Introduction

The accumulation of carbon dioxide (CO_2_) in the atmosphere due to fossil fuel burning has increased global gross primary production (GPP) and decreased the isotopic composition of atmospheric CO₂ (δ^13^CO_2_) – the ratio of the heavier (^13^C) to the lighter (^12^C) stable carbon isotope of atmospheric CO_2_ – over the past century, a phenomenon known as the Suess effect^1^. While changes in GPP can influence carbon isotope discrimination (Δ¹³C) during photosynthesis, the relationship is not strictly linear and depends on environmental and physiological conditions^2^, which means shifts in plant productivity may affect δ¹³CO₂ in complex ways. Atmospheric measurements show that δ^13^CO_2_, expressed as the normalized ratio of ¹³C to ¹²C compared to a standard, has declined by 0.027‰ per year over the period from 1978 to 2014^3,4^. However, the observed δ^13^CO_2_ decrease is apparently smaller than expected when accounting for land and ocean carbon cycling and uptake in a simple calibrated model, which predicts a decline of about 0.032‰ per year^3^. To explain this shortfall, Δ^13^C of the terrestrial biosphere should have increased by 0.014 ± 0.007‰ per ppm of CO_2_ increase^3^. Whether a Δ^13^C change of this magnitude is consistent with actual terrestrial carbon fluxes remains uncertain.

Global Δ^13^C estimates from long-term tree-ring measurements do not show the increase in the Δ^13^C of C_3_ plants postulated by ref. ^3^. Although the Δ^13^C of C_3_ plants as recorded by tree rings has been variable across sites (increasing, decreasing, or unchanging^2,5,6^), globally it has remained roughly constant^2^. It is possible that post-photosynthetic fractionation processes^2,7^ and intrinsic age-related changes in tree development over their lifespan, such as tree height^8,9^ could affect inferences of long-term Δ^13^C trends from tree rings. However, these effects are not well understood or quantified. No alternative source of data for C_3_ plants are currently available. The mean residence time of C_4_ plant-derived carbon in the biosphere is generally shorter than that of C_3_ plant-derived carbon^10,11^ and there is no direct evidence of changes in Δ^13^C of C_4_ plants. Since atmospheric measurements reflect large-scale changes in vegetation dynamics while in situ measurements only record ecophysiological adjustments from the ecosystems studied, it is challenging to reconcile measurements on the ground with estimates from the atmosphere. Current models linking carbon fluxes and stocks between land and the atmosphere are complex, making comparison difficult. Simple modeling approaches are needed to determine recent changes in global GPP and associated Δ^13^C, and to disentangle the contribution from C_3_ and C_4_ plants to the observed atmospheric trends.

The global distribution of C_3_ and C_4_ plants reflects their divergent responses to climate as well as human activities via cropping and land management^11,12^. C_3_ plants include cool-climate grasses, most shrubs, and nearly all trees^13^, while C_4_ plants generally dominate in warm-climate grasslands and savannas. C_4_ plants possess a unique set of adaptations making them more competitive than C_3_ plants in warm, arid, and high-light environments^14,15^, primarily via reduced rates of photorespiration. In contrast, C_3_ photosynthesis is stimulated at high atmospheric CO_2_ concentrations. This is known as the CO_2_ fertilization effect and confers an advantage over C_4_ photosynthesis under elevated CO_2_^16^.

Variations in Δ^13^C are closely related to environmentally driven changes in the stomatal limitation of photosynthesis, expressed as the ratio of leaf-internal to ambient partial pressures of CO_2_. Δ^13^C also depends on the pathway of carbon assimilation. Isotopic fractionation during the diffusion of CO_2_ through the stomata primarily influences Δ^13^C in C_4_ plants, while fractionation during Rubisco carboxylation has a stronger imprint on Δ^13^C in C_3_ plants, resulting in C_3_ plants being depleted in ^13^C compared to C_4_ plants^17–19^. Knowledge of the different isotopic signatures of C_3_ and C_4_ photosynthetic pathways and of their relative coverage across the globe can be used to estimate average δ^13^C across terrestrial environments, and so global Δ^13^C.

Several models of the distribution of C_3_ and C_4_ plants have been proposed. By far the most widely used C_4_ distribution map in ecophysiological research and land-surface modeling is the one developed by ref. ^20^ based on an approach published in ref. ^11^—see for example,^21–24^. However, this map is static, implying constancy over time. More recent work has considered the differential responses of C_3_ and C_4_ plants to recent environmental changes, based on an optimality model^12^. The derived map^12^ indicates that the global fraction of C_4_ plants has decreased over 2001–2019 due to a decrease in the natural abundance of C_4_ grasses with elevated CO_2_, even as the area of C_4_ crops increased. Such a shift would impact trends in global GPP and Δ^13^C. Reference ^2^ suggested that the lower-than-expected decrease in global δ^13^CO_2_ observed in atmospheric measurements (attenuation of the Suess effect)^3^ might be explained by changes in the abundance and distribution of C_3_ and C_4_ plants. In contrast, ref. ^3^ suggested that any change in C_3_/C_4_ distribution would have a negligible impact on atmospheric δ^13^CO_2_, given the higher carbon turnover rate in C_4_ than C_3_ plants—implying a dominant control on atmospheric δ^13^CO_2_ by C_3_ photosynthesis.

Here we propose a new C_3_/C_4_ distribution model based on the well validated, optimality-based P model^25–27^ to test refs. ^2^, ^3^ hypotheses (hereafter denoted as Lavergne2022 and Keeling2017, respectively) and to determine whether changes in C_3_ and C_4_ plant distributions could explain the observed decrease in atmospheric δ^13^CO_2_ over the period from 1982 to 2016 (see workflow in Supplementary Fig. 1). We compiled a large global dataset of stable carbon isotope measurements from leaves^28^ and soils^29^ to evaluate model predictions of photosynthetic Δ^13^C in C_3_ and C_4_ plants and C_3_/C_4_ fractions, respectively. δ^13^C_soil_ is a good indicator of local changes in the abundance of C_3_ and C_4_ plants because of the contrasting isotopic signatures of the two photosynthetic pathways. We estimated recent changes in the abundance and distribution of C_3_ and C_4_ plants, GPP and Δ^13^C in response to environmental changes and compared them with those based on the C_4_ distribution maps of ref. ^20^ and ref. ^12^ (hereafter denoted as Still2009 and Luo2014, respectively). We performed an attribution analysis to determine the relative contributions of environmental drivers to the changes in the fraction of C_4_ plants, GPP, and Δ^13^C. Finally, we used a simple carbon-cycle box model^30,31^ to determine whether changes in C_3_ and C_4_ biomass, weighted by their relative fractions and carbon turnover times, could explain the magnitude of the observed decrease in atmospheric δ^13^CO_2_.

## Results

**Fig. 1:**
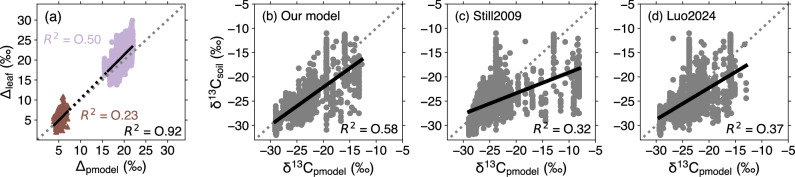
Predictive skill of the model to reproduce leaf and soil stable carbon isotope data. **a** Comparison between predicted and observed Δ^13^C for C_3_ (purple) and C_4_ (brown) plants with associated coefficients of determination (R^2^). The R^2^ for all (C_3_ + C_4_) plants is shown in black. Comparison between predicted and observed δ^13^C_soil_ for **b** our C_3_/C_4_ competition map, **c** the global C_4_ map of Still2009, and **d** Luo2024, with associated coefficients of determination (R^2^).

The skill of the model to predict Δ^13^C for C_3_ and C_4_ plants was good with coefficients of determination (R^2^) averaging 0.50, 0.23 and 0.92, respectively for C_3_, C_4_ and total (C_3_ and C_4_) plants (Fig. 1a). However, the model underestimated the leaf-derived variability of Δ^13^C (standard deviation = 1.56‰ versus 2.63‰ for C_3_ plants, and 0.60‰ versus 1.32‰ for C_4_ plants, respectively for model and observations). The model reproduced 58% of the variability of the global soil isotopic δ^13^C_soil_ records (R^2^ = 0.58; Fig. 1b), an improvement over the simulations using the C_4_ maps of Still2009 and Luo 2024 (R^2^ = 0.32 and 0.37, respectively; Fig. 1c, d).

**Fig. 2:**
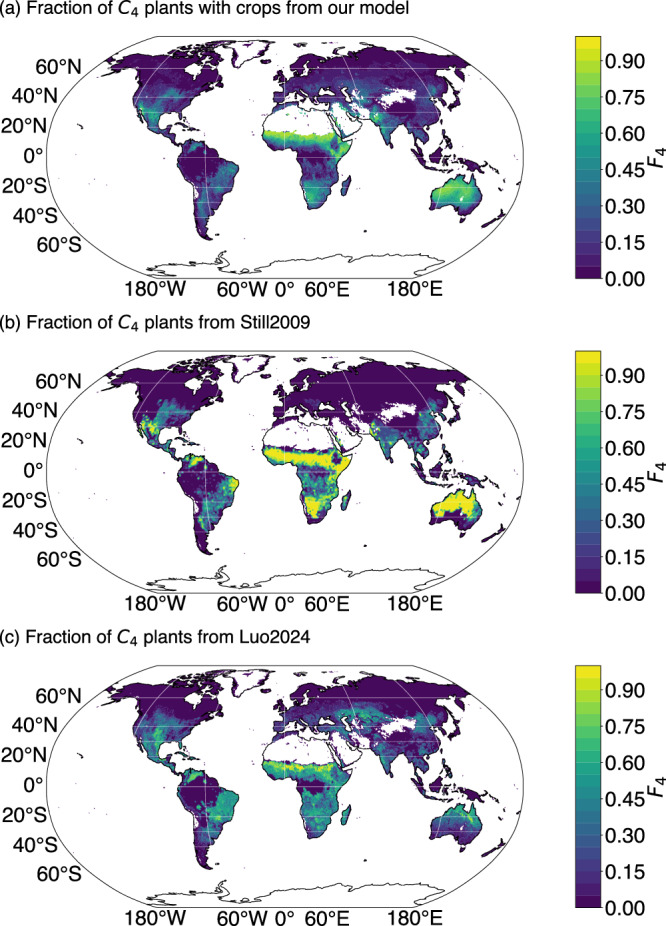
Predicted fraction of C_4_ plants across the globe over 2001–2016. Model predictions using our simple C_3_/C_4_ approach, including both natural grasslands and croplands (**a**). Global C_4_ distribution maps from Still2009 (**b**) and Luo2024 (**c**).

**Fig. 3:**
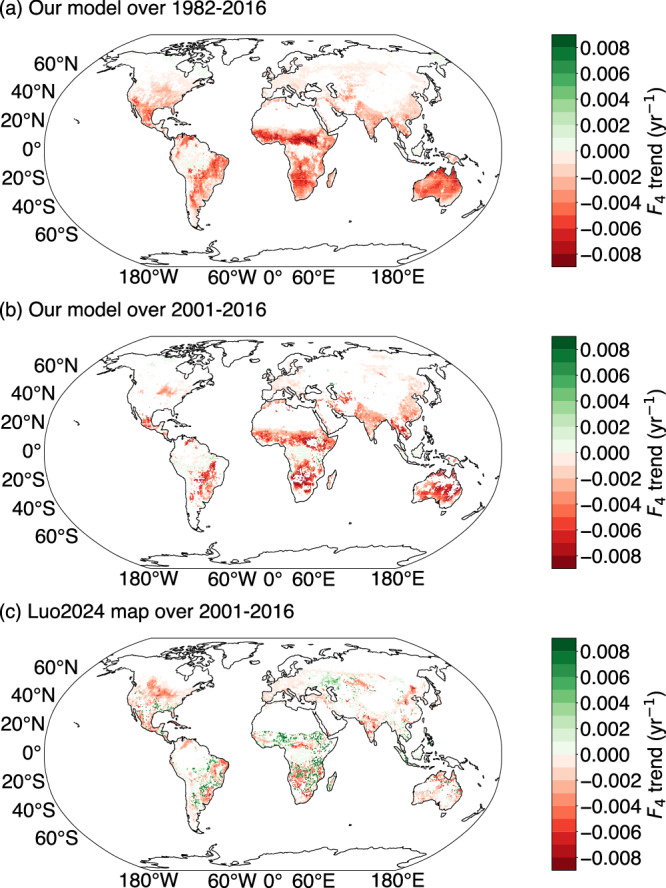
Temporal trend in the global fraction of C_4_ plants (*F*_4_, in yr^-1^). Trend in *F*_4_ from our C_3_/C_4_ map over 1982–2016 (**a**) and 2001–2016 (**b**). Trend in *F*_4_ from Luo over 2001–2016 (**c**).

**Fig. 4:**
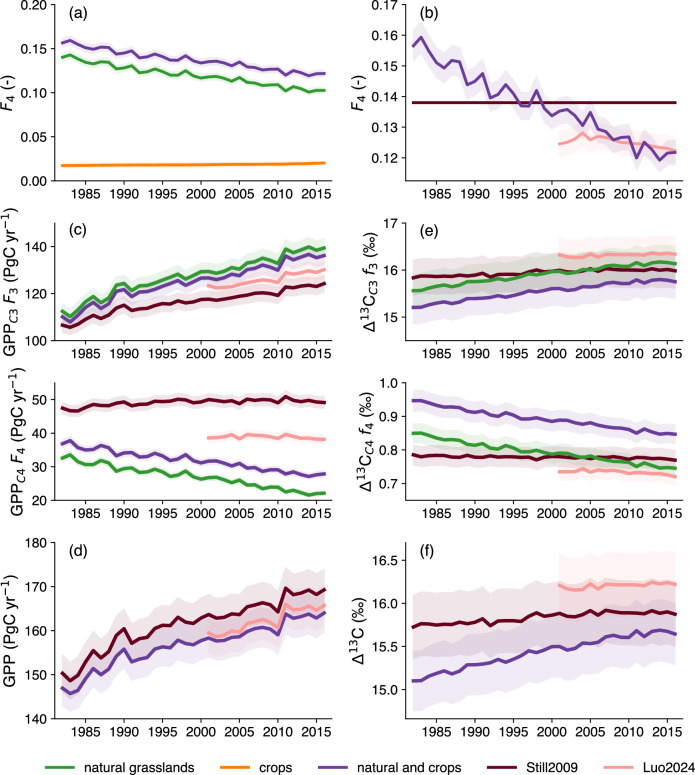
Global changes in the fraction of C_4_ plants, GPP and Δ^13^C over 1982–2016. Fraction of C_4_ plants (*F*_4_) from our model for (**a**) natural grasslands (green), crops (orange) and both natural grasses and crops (violet) and (**b**) total *F*_4_ compared to that from Still2009 (brown) and Luo2024 (light pink). GPP weighted by the fraction of C_3_/C_4_ plants (in PgC yr^−1^) for natural grasslands (green), crops (orange) and both natural grasses and crops (violet) in C_3_ (**c**) and C_4_ (**d**) plants and (**e**) for all plants (including C_3_ and C_4_ plants; Eq. 3c) using our maps and those. Δ^13^C weighted by the fraction of C_3_/C_4_ plants (in ‰) for natural grasslands (green), crops (orange) and both natural grasses and crops (violet) in C_3_ (**f**) and C_4_ (**g**) plants and (**h**) for all plants (including C_3_ and C_4_ plants; Eq. 4) using our map, Still2009 and Luo2024. The light shade is the uncertainty calculated as the 95% confidence interval of the simulated mean (**a**, **b**, **f**, **g**, **h**) or sum (**c**–**e**) plus the sum of uncertainties from the input data (assumed to be ±2% of the global average values). The uncertainty range of the C_4_ cropland area derived from LUHv2-2019 (orange) is assumed to be ±10% of the global average values following Luo.

The predicted fraction of C_4_ plants (*F*_4_) was large in hot and dry regions including subtropical Africa, the southern part of North America, northeastern Brazil and northern Australia, but low in cold and temperate regions (Fig. 2a). Similar patterns of spatial variation were shown in Still2009 and Luo2024 (Fig. 2b, c), but were more pronounced in Still2009 than in the other two maps. Predicted *F*_4_ tended to decrease over the period studied in most regions with *F*_4_ > 5% (Fig. 3a), but to increase slightly in southern equatorial regions (0–20°S) in central Eurasia and high latitudes of North America (Fig. 3b). The predicted *F*_4_ decrease was greater than in Luo2024 over their common 2001–2016 period (Fig. 3b, c). Luo2024 showed increases in *F*_4_ in more regions than in our map (Fig. 3c). Globally, according to our model, *F*_4_ decreased from 14.1 to 10.3% for natural grasslands but increased from 1.7 to 2.0% for crops between 1982 and 2016. This resulted in a decrease of global *F*_4_ (considering both natural grasslands and crops) from 15.8 to 12.2% over the same period (Fig. 4a), and from 13.6 to 12.2%. Global *F*_4_ was 13.8% in Still2009 but decreased over 2001–2016 from 12.5 to 12.3% in Luo2024 (Fig. 4b). *F*_4_ decreased by 0.001% yr^−1^ (p < 0.001) in our analysis, but at a slower rate for Luo2024 (<0.001% yr^−1^, p < 0.01). The global average *F*_4_ over the common 2001–2016 period was 12.7% using our model, similar to Luo2024 (12.5%).

From 1982 to 2016, while the predicted gross primary production (GPP) per unit land area for C_3_ plants (GPP_C3_) increased across the globe, GPP for C_4_ plants (GPP_C4_) decreased in line with the *F*_4_ decrease (Supplementary Fig. 2a, b). Total GPP, including both C_3_ and C_4_ photosynthesis, increased almost everywhere, but decreased in South America around 30°S, in northwestern Australia and in Africa around 20°N and 20°S (Supplementary Fig. 2c). Globally, GPP_C3_ increased at a rate of 0.75 ± 0.06 PgC yr^–2^ and GPP_C4_ decreased by 0.28 ± 0.02 PgC yr^–2^ (Fig. 4c), resulting in an increase of total GPP (including C_3_ and C_4_ natural grasslands and crops) by 0.47 ± 0.05 PgC yr^−2^—equivalent to an increase of 16.5 ± 1.8 PgC yr^–1^—during 1982–2016 (Fig. 4d). These estimates differ from those obtained using the fixed C_4_ distribution map of Still2009, where GPP increased at a faster rate of 0.53 ± 0.06 PgC yr^–2^—equivalent to an increase of 18.6 ± 2.1 PgC—with GPP_C3_ and GPP_C4_ increasing by 0.48 ± 0.04 PgC yr^–2^ and 0.07 ± 0.02 PgC yr^–2^ respectively (Fig. 4d). Over the 2001–2016 period, the rate of increase in GPP was slightly lower using our map than that of Still2009 or Luo2024: 0.45 ± 0.11 compared to 0.46 ± 0.12 and 0.50 ± 0.13 PgC yr^–2^, respectively. The mean GPP was similar using our map and that of Luo2024 (Fig. 4d). The average contribution of C_4_ photosynthesis to global GPP over 2001–2016 was around 16.2% for natural grasslands, and 18.5, 18.6, and 17.5% for total C_4_ plants (including crops) based on our map and the Still2009 and Luo2024 maps, respectively.

Predicted Δ^13^C increased for C_3_ plants (Δ^13^C_C3_) but decreased for C_4_ plants (Δ^13^C_C4_) from 1982 to 2016 (Supplementary Fig. 3a, b), resulting in an increase of total Δ^13^C, including both C_3_ and C_4_ photosynthesis, almost everywhere (Supplementary Fig. 3c). Globally, when using a constant *F*_4_ from the Still2009 map, Δ^13^C increased by 0.005 ± 0.001‰ yr^−1^ for C_3_ plants over 1982–2016 (p < 0.001) equivalent to 0.003 ± 0.001‰ per ppm increase of CO_2_, while Δ^13^C for C_4_ plants stayed broadly constant, decreasing by <0.001‰ yr^–1^ (p < 0.001) equivalent to <0.001‰ ppm^–1^ (Fig. 4e), resulting in a total Δ^13^C (including both C_3_ and C_4_ plants) increase of 0.005 ± 0.001‰ yr^–1^ (Fig. 4f) equivalent to 0.003 ± 0.001‰ ppm^–1^. However, when considering the global decrease in *F*_4_ over 1982–2016 using our C_4_ map, Δ^13^C in C_3_ plants increased at a faster rate (0.018 ± 0.001‰ yr^–1^, equivalent to 0.010 ± 0.001‰ ppm^–1^, p < 0.001), while Δ^13^C in C_4_ plants decreased by 0.003 ± 0.001‰ yr^–1^, equivalent to 0.002 ± 0.001‰ ppm^–1^ (p < 0.001), leading to a total Δ^13^C increase of 0.017 ± 0.001‰ yr^–1^, equivalent to 0.010 ± 0.001‰ ppm^–1^ (p < 0.001). Over the common period 2001–2016, global Δ^13^C increased by 0.004 ± 0.002‰ yr^–1^, equivalent to 0.002 ± 0.001‰ ppm^–1^ (p < 0.01) using Luo2024, and 0.014 ± 0.005‰ yr^−1^, equivalent to 0.007 ± 0.001‰ ppm^–1^, using our map.

**Fig. 5:**
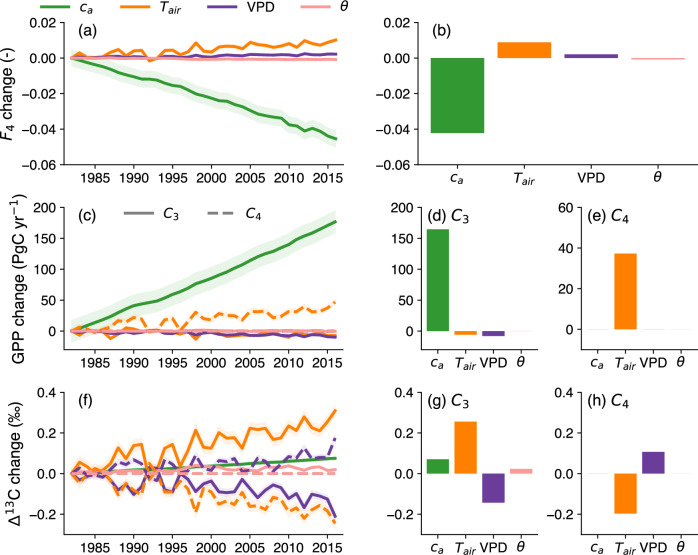
Global averaged change in the impact of environmental drivers on F_4_, GPP, and Δ^13^C. Temporal changes of the impact of atmospheric CO_2_ concentrations (*c*_a_), daytime air temperature (*T*_air_), vapor pressure deficit (VPD), and soil moisture (θ) on *F*_4_ (**a**), and GPP (**c**) and Δ^13^C (**f**) for C_3_ and C_4_ plants over 1982–2016. **b**, **d**, **e**, **g**, **h** Global average of the individual impacts over the last 5 years (2012–2016). In (**a**, **c**, **f**), the light shade represents the 95% confidence interval of the simulated mean.

The decrease in global *F*_4_ was mainly driven by the increase in atmospheric CO_2_ concentration (*c*_a_), followed by increasing daytime air temperature (*T*_air_) and, to a lesser extent, by increasing daytime vapor pressure deficit (VPD) and soil moisture (*θ*) (Fig. 5a, b). Compared to *c*_a_, *T*_air_ and VPD, which showed spatially more-or-less homogeneous impacts on *F*_4_, the *θ* contribution to *F*_4_ was heterogeneous across the globe (Supplementary Fig. 4), reflecting heterogeneity in hydroclimatic changes (Supplementary Fig. 5). As expected, rising *c*_a_ was the major contributor of GPP increase for C_3_ plants, while rising *T*_air_ increased GPP in C_4_ plants (Fig. 5c–e). *T*_air_ was the most important driver of Δ^13^C for both C_3_ and C_4_ plants, followed by VPD. Δ^13^C in C_3_ plants increased most with higher *T*_air_, followed by *c*_a_ and to a lesser extent *θ*, but decreased with higher VPD (Fig. 5f, g). In contrast, the decrease in Δ^13^C for C_4_ plants was mainly driven by rising *T*_air_, while the increase in VPD attenuated this decrease (Fig. 5f, h).

**Fig. 6:**
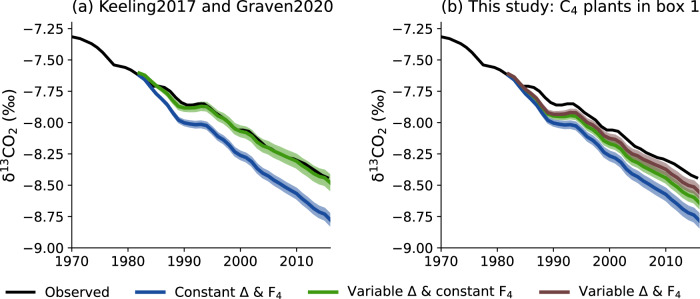
Observed versus predicted δ^13^CO_2_ (‰) estimated from a simple carbon cycle model with three biosphere boxes. **a** Original model configuration predicting δ^13^CO_2_ with simple (blue) and CO_2_-driven (green) Δ^13^C as in ref. ^3^ and ref. ^30^—denoted Keeling2017 and Graven2020, respectively. **b** Model predictions when box 1 represents only C_4_ plants, while boxes 2 and 3 are populated only by C_3_ plants. In blue is the simulation when both Δ^13^C and *F*_4_ are constant. In green is the prediction when Δ^13^C is modeled as in Equations S11–S13 with constant *F*_4_ and *F*_3_. In brown is the simulation when Δ^13^C, *F*_4_, and *F*_3_ vary.

Running the simple carbon cycle box model, in its original version^30^, we were able to reproduce the results of ref. ^3^: a larger-than-observed decrease in atmospheric δ^13^CO_2_ when assuming a constant Δ^13^C (18‰), but consistent observed and predicted trends when using a varying Δ^13^C driven by increases in atmospheric CO_2_ (Fig. 6a). Assuming a constant *F*_4,_ and with the simplifying assumption that box 1 of the model (with small biomass and fast turnover) represents C_4_ vegetation while boxes 2 and 3 (with intermediate to high biomass and intermediate to low turnover) include only C_3_ vegetation, we predicted a faster-than-observed decrease in atmospheric δ^13^CO_2_ (Fig. 6b). The difference between observations and predictions was greater when we assumed a constant Δ^13^C (equal to 6‰ for C_4_ plants and 18‰ for C_3_ plants) than when we let Δ^13^C vary annually using our global model outputs (difference between observed and predicted Δ^13^C of around 0.30 versus 0.16‰ in 2012–2016). When accounting for both varying Δ^13^C and *F*_4_, our predicted δ^13^CO_2_ values were closer to the observed trends, but they did not fully reproduce the magnitude of the decrease (the difference was around 0.08‰ over 2012–2016). Thus, by accounting for both changes in Δ^13^C and *F*_4_ and for differences in biomass and carbon turnover for C_3_ and C_4_ plants, the difference between observed and predicted trends in atmospheric δ^13^CO_2_ was only slightly reduced.

## Discussion

We estimated the global fraction of C_4_ plants (*F*_4_) based on a simple optimality modeling approach driven by climate reanalyses and remote sensing observations to quantify spatiotemporal changes in *F*_4_, gross primary production (GPP), and land carbon isotopic discrimination (Δ^13^C) over the period from 1982 to 2016. We also used a simple carbon-cycle box model to determine whether the observed magnitude of the decrease in global atmospheric δ^13^CO_2_ could be explained by recent changes in the global abundance and distribution of C_3_ and C_4_ plants, as suggested by Lavergne2022.

### Spatiotemporal variations in predicted *F*_4_ and differences among approaches

The spatial patterns of *F*_4_ predicted by our approach are quite similar to those from Still2009 and Luo2024 over the common period (2001–2016). However, Still2009 tends to overestimate *F*_4_ in sub-Saharan and southern Africa and northern Australia compared to our study and that of Luo2024—as also indicated by the higher predicted than observed δ^13^C_soil_ values found in Still2009 for these regions (Fig. 1c). C_4_ plants are important components of African savannas, grasslands, and shrubs^32^ and dominate grasslands in the subtropical northeast of Australia^33,34^, but they are probably not as overwhelmingly dominant in these regions as the Still2009 map suggests. Both our model and that of Luo2024 capture the relative abundance of C_3_ and C_4_ plants in this region. No soil isotope data were available in the dry northeast of Brazil (the Caatinga region) to evaluate the maps; however, the region is known to have a high diversity of both C_3_ and C_4_ plants^35,36^. The Still2009 estimate that more than 80% of Caatinga vegetation follows the C_4_ pathway is thus probably too high, and the co-dominance of C_3_ and C_4_ plants suggested by our map and Luo (*F*_4_ around 0.5–0.6) seems more realistic.

The Luo2024 map predicts that on average, from 2001 to 2016, C_4_ plants, including natural grasslands and crops, occupied around 12.5% of the global land surface (excluding deserts and ice-covered lands) and contributed 17.5% of global photosynthesis. These values are slightly lower than those given in the original article (17.5% and 19.5%, respectively) because we considered a larger global land area. Our model estimates are similar to those of Luo2024. We predict that the fraction of C_4_ plants is 12.7%, contributing 18.5% of global photosynthesis. The value is consistent with previous estimates (18–23%^11,37,38^) and higher than the ensemble mean of dynamic global vegetation models (14 ± 13%^12^). The predicted decrease in global *F*_4_ is greater using our map than using Luo2024, probably because we predicted a decrease in the fraction of C_4_ in more regions.

### Spatiotemporal changes in GPP and Δ^13^C driven by *F*_4_ and implications for atmospheric δ^13^CO_2_ variations

The predicted increase in annual total GPP considering both C_3_ and C_4_ photosynthesis (16.5 ± 1.8 PgC) from 1982 to 2016 falls within the range of recently published values^21,39^. The 31 ± 5% increase in GPP over 1900–2010 reconstructed from ice-core records of carbonyl sulfide^40^ implies that annual GPP may have increased by around 14 ± 5% (equivalent to 17.2 ± 1.5 PgC) over 1982–2016^21^. Using a light-use efficiency model driven by remote sensing observations^41^, suggested an increase in the global GPP of 0.27 ± 0.02 Pg C yr^–2^ (or 9.5 ± 0.7 PgC yr^−1^, i.e., a 6.1% increase using as reference the mean 1982-1983 values of 115 Pg C yr^−1^) over the period from 1982 to 2015. The spatial distribution of GPP changes highlighted in Fig. S2c is also consistent with that predicted by ref. ^21^, i.e., an increase in GPP in regions where the CO_2_ effect on C_3_ photosynthesis is the largest contributor to the change in GPP^39^, such as tropical and European forests. It differs from ref. ^41^, who suggested a decrease in GPP in large parts of the tropics, but that study ignored the CO_2_ fertilization effect. Although net carbon uptake in parts of the Amazon rainforest has declined over the past three decades due to increased carbon losses from tree mortality^42^, recent estimates of global forest land changes suggest GPP increased in most forests over the past decade^43^, in line with our predictions.

When assuming a constant *F*_4_, predicted global Δ^13^C including both C_3_ and C_4_ plants increased by 0.003 ± 0.001‰ per ppm increase of CO_2_ over the 1982–2016 period, consistent with other estimates that ignored changes in the C_3_/C_4_ fraction^2^. However, when the global decrease in *F*_4_ over the study period is accounted for, global Δ^13^C increased by 0.010 ± 0.001‰ ppm^–1^ due to the increase in Δ^13^C of C_3_ plants (and also the slight decrease in Δ^13^C of C_4_ plants). Including crops into this analysis has a minimal effect on Δ^13^C and does not significantly change the magnitude of the global Δ^13^C trends.

Over their common period (2001–2016), the magnitude of global Δ^13^C increase predicted using our *F*_4_ map is larger than using Luo2024, despite their relative agreement in predicting global *F*_4_ and GPP increase over the recent years. The two maps agree from the late 2000s onwards. The difference could be due to an overestimation of the fraction of C_3_ plants (*F*_3_) across the globe in our map, exacerbating the increasing global Δ^13^C trend. We assumed that the sum of *F*_3_ and *F*_4_ in each land grid point (excluding deserts and snow/glacial lands) is always equal to 1, but this might be an overestimate, as some areas may be covered by other types of land surface.

Using the simple carbon cycle box model, we show that accounting for Δ^13^C variations alone—contrary to Keeling2017—cannot explain the observed reduction of the Suess effect in atmospheric δ^13^CO_2_. However, predicted global changes in the abundance and distribution of C_3_ and C_4_ plants only slightly reduce the differences between observations and predictions during 2012–2016. The combined effects of increasing global Δ^13^C and decreasing fraction of C_4_ plants reduce the difference from 0.30 to 0.08‰. Thus—contrary to Lavergne2022—our analysis cannot explain the full magnitude of the decrease in atmospheric δ^13^CO_2_, indicating a need to consider other drivers of the isotopic signature of atmospheric CO_2_. Uncertainties in biosphere processes—especially isotopic fractionation during post-photosynthetic pathways and soil respiration, and carbon residence times in soils—as well as ocean–atmosphere exchanges and fossil fuel emissions, can cause discrepancies between simulated and observed atmospheric δ^13^CO_2_^30,44^.

### Limitations of our analysis

The approach to C_3_/C_4_ distribution by Luo2024 is based on a model predicting influences of atmospheric CO_2_, water stress (soil moisture and vapor pressure deficit), and nitrogen availability on both C_3_ and C_4_ photosynthesis^45^. Our model is simpler; it assumes that the impacts of elevated CO_2_ and water stress are important only for C_3_ photosynthesis (Supplementary Figs. 11 and 12) and neglects nitrogen availability effects. Nonetheless, our attribution analysis suggests patterns of variability of *F*_4_ with elevated CO_2_ and water stress (Fig. 5) similar to those in Luo2024. Global *F*_4_ and GPP values predicted with our *F*_4_ model over the recent years are in line with those using the Luo2024 map over their common period (2001–2016). Finally, when comparing measured and predicted δ^13^C_soil_, our simple model provides better predictions of δ^13^C_soil_ than Luo2024 (*R*^2^ = 0.58 versus 0.37), giving us confidence in our predictions.

We considered the photorespiratory effect on Δ^13^C trends for C_3_ plants, but not additional effects (notably the effect of mesophyll conductance), whose importance is debated. Keeling2017 suggested a contribution from the mesophyll conductance effect to the globally increasing Δ^13^C trends of 0.006 ± 0.003‰ per ppm increase of CO_2_, which would induce a higher Δ^13^C trend in C_3_ photosynthesis than predicted here: 0.009 ± 0.003‰ ppm^−1^ (as opposed to 0.003 ± 0.001‰ ppm^−1^) over 1982–2016. On the other hand, Lavergne2022 argued that the mesophyll effect could reduce Δ^13^C values and trends by around –0.001 ± 0.001‰ ppm^−1^, so the magnitude of the increase in Δ^13^C reported here would then be overestimated. This argument relied on the assumption that the ratio of stomatal to mesophyll conductance is independent of environmental factors, leading to an optimal ratio of the chloroplastic to ambient CO_2_^27^. This is probably oversimplified; however, the controls of mesophyll conductance are not fully understood.

Finally, when we tested the simple carbon-cycle box model for its sensitivity to changes in the abundance of C_3_ and C_4_ plants on atmospheric δ^13^CO_2_, we only incorporated our global Δ^13^C estimates for C_3_ and C_4_ plants as model inputs, modulated by changes in their relative abundance, carbon use efficiency, and turnover. The standard biosphere carbon model embedded in the box model does not differentiate C_3_ and C_4_ photosynthesis and allows the sizes of all three land reservoirs to grow due to CO_2_ fertilization. Although we removed the CO_2_ fertilization effect from the first box (C_4_ photosynthesis), these simplifications of the drivers of C_3_ and C_4_ photosynthesis may have biased estimates of net primary production (NPP) and increased uncertainties in modeled carbon dynamics. We also made some assumptions regarding carbon turnover values for the different plant types examined, based on the published parameterization of the carbon cycle box model^30^. For instance, we assume that the carbon turnover for C_4_ herbaceous (box 1) is about 10 times lower than that of C_3_ herbaceous (box 2). Although there is ample evidence showing that the turnover time of C_4_-derived soil carbon is substantially shorter than that of C_3_-derived soil carbon^46^, carbon turnover may still vary across regions. The extent of these variations and potential underlying drivers are still not well constrained.

Regardless of the simplifications in our analysis, however, our results imply that neither of the tested hypotheses^2,3^ provide a comprehensive explanation for the observed trend in atmospheric δ^13^CO_2_. Recent shifts in global Δ^13^C and the abundance and distribution of C_3_/C_4_ plants can only partially explain the magnitude of the observed decrease in δ^13^CO_2_. Factors other than changes in species abundance and distribution must influence carbon isotope flux exchanges. Further analyses are needed to determine their nature and quantify their contribution to the Suess effect.

## Methods

### Modeling approach based on optimality principles

We used the P model^25–27^, a light-use efficiency model based on eco-evolutionary optimality principles, to simulate ecosystem gross primary production (GPP) and carbon isotope discrimination (Δ^13^C). We also developed a simple scheme based on the P model to predict the share of C_4_ plants in the total GPP (see Supplementary Note 1 for more details on the model and workflow in Supplementary Fig. 1). We converted the potential share of C_4_ plants in the total GPP to fraction of C_4_ plants (*F*_4,pot_) using the emergent constraint in ref. ^12^ as the share of C_4_ plants divided by 1.13.

Since the total fractions of C_3_ and C_4_ biomass consist of natural ecosystems (trees, grasses) and crops, we estimated the C_4_ fraction of natural ecosystems (*F*_4,nat_) by removing the fraction of human managed areas (C_3_ and C_4_ crops and urban areas) from *F*_4,pot_:

1 $${F}_{4,{nat}}={F}_{4,{pot}}-\left({F}_{3,{crops}}+{F}_{4,{crops}}\right){-F}_{{urban}}$$

We then calculated the total fraction of C_4_ and C_3_ plants (*F*_4,tot_ and *F*_3,tot_) considering natural ecosystems (*F*_4,nat_ and *F*_3,nat_) and crops (*F*_4,crops_ and *F*_3,crops_) as:

2a $${F}_{4,{tot}}={F}_{4,{nat}}+{F}_{4,{crops}}$$

2b $${F}_{3,{tot}}={1-F}_{4,{tot}}={F}_{3,{nat}}+{F}_{3,{crops}}$$

We estimated C_3_, C_4_ and total (C_3_ + C_4_) GPP from their respective potential GPP (${{GPP}}_{C3,{pot}}$ and ${{GPP}}_{C4,{pot}}$) as:

3a $${{GPP}}_{C3}={{GPP}}_{C3,{pot}}{F}_{3,{tot}}$$

3b $${{GPP}}_{C4}={{GPP}}_{C4,{pot}}{F}_{4,{tot}}$$

3c $${{GPP}}_{{tot}}={{GPP}}_{C3}+{{GPP}}_{C4}$$

We aggregated GPP for C_3_, C_4_, and all (C_3_ + C_4_) plants from each grid cell, weighted by grid-cell area for each prediction with each C_4_ map, and compared their respective temporal changes (GPP, in PgC yr^−1^).

We then estimated the land Δ^13^C assuming that the biosphere is composed of three terrestrial components (C_4_ herbaceous, C_3_ herbaceous and C_3_ woody) with different carbon turnover times (${{{\rm{\tau }}}}$, year) and carbon use efficiencies (CUE, unitless; the ratio of net to gross primary productivity, NPP/GPP):

4 $${\Delta }^{13}C=\frac{{f}_{4,{herb}}\,{\Delta }_{4}\,{{{{\rm{\tau }}}}}_{4,{herb}}+{f}_{3,{herb}}\,{\Delta }_{3}{{{{\rm{\tau }}}}}_{3,{herb}}+{f}_{3,{woody}}\,{\Delta }_{3}{{{{\rm{\tau }}}}}_{3,{woody}}}{{{{{\rm{\tau }}}}}_{4,{herb}}+{{{{\rm{\tau }}}}}_{3,{herb}}+{{{{\rm{\tau }}}}}_{3,{woody}}}$$

with ${{{{\rm{\tau }}}}}_{4,{herb}},\,{{{{\rm{\tau }}}}}_{3,{herb}},{{{{\rm{\tau }}}}}_{3,{woody}}$ the carbon turnover times and *f*_4,herb_, *f*_3,herb_ and *f*_3,woody_ the fractions adjusted by CUE (CUE_4,herb_, CUE_3,herb_ and CUE_4,woody_) for C_4_ herbaceous, C_3_ herbaceous and C_3_ woody plants respectively.

4a $${f}_{4,{herb}}={F}_{4,{tot},t0}-\left({F}_{4,{tot},t0}-{F}_{4,{tot}}\right){\,{CUE}}_{4,{herb}}$$

4b $${f}_{3,{herb}}={F}_{3,{tot},t0}-\left({F}_{3,{tot},t0}-{F}_{3,{tot}}\right){\,{CUE}}_{3,{herb}}$$

4c $${f}_{3,{woody}}={F}_{3,{tot},t0}-\left({F}_{3,{tot},t0}-{F}_{3,{tot}}\right)\,{{CUE}}_{3,{woody}}$$

${F}_{4,{tot},t0}$ and ${F}_{3,{tot},t0}$ are the fractions of C_4_ and C_3_ plants, respectively, at time *t*0 - the first year of the records (1982).

Δ_4_ and Δ_3_ are the carbon isotope discrimination of C_4_ and C_3_ plants, respectively (see Supplementary Note 1), calculated for each month (*mth*) and weighted by each month’s C_3_ and C_4_ GPP to obtain annual averages of Δ_3_ and Δ_4_:

5 $${\Delta }_{x,{yr}}=\sum\limits_{t}\frac{{\Delta }_{x,{mth}}\times {{GPP}}_{x,{mth}}}{{\sum }_{t}{{GPP}}_{x,{mth}}}$$

with *x* being the plant type (C_3_ or C_4_) selected.

On average, biomes dominated by C_4_ plants have both higher carbon turnover rates and lower biomass than those dominated by C_3_ plants^46–48^, so their relative contribution to land ∆^13^C is expected to be lower than that of C_3_ plants. ${{{\rm{\tau }}}}$ values estimated as the ratio of carbon stocks in vegetation and soils to NPP from TRENDYv12 model outputs^49^ vary strongly among models. For instance, global average ${{{\rm{\tau }}}}$ (median ± standard deviation) are 8.7 ± 11.2, 22.4 ± 34.2 and 15.3 ± 27.5 year for C_4_ grasses, 33.8 ± 33.3, 40.5 ± 81.1 and 534.3 ± 217.7 year for C_3_ grasses, and 561.6 ± 173.7, 616.0 ± 224.3, and 641.3 ± 181.2 year for C_3_ woody, respectively for CABLE-POP, CLASSIC, and ORCHIDEE models. Given the large uncertainties in these estimates and to ensure consistency with our whole approach, we use the same range of ${{{\rm{\tau }}}}$ values as in a carbon cycle box model parameterization when the CO_2_ fertilization effect is on for C_3_ plants^30^ (${{{\rm{\tau }}}}$_4,herb_ = 2.4 ± 0.3 year, ${{{\rm{\tau }}}}$_3,herb_ = 24.4 ± 6.5 year, and ${{{\rm{\tau }}}}$_3,woody_ = 299.4 ± 144.5 year; see also Supplementary Table 1). These values are at the lower end of the estimates from the three TRENDYv12 models but are consistent with the relative differences in τ between C_4_ herbaceous, C_3_ herbaceous and C_3_ woody plants (very fast, intermediate, and slow, respectively).

Non-forest biomes, including grasslands, shrublands, and crops, tend to have higher CUE than forests (0.46  ±  0.11 versus 0.41  ±  0.11), except for C_4_ grass-dominated savanna ecosystems, which show a lower CUE (0.32  ±  0.12)^50^. However, the contribution of C_3_ and C_4_ plants in grasslands, shrublands, and crops, and their relative CUEs remain unclear. Here, we use the average CUE values reported in reference 46, assuming that C_4_ biomass is a mixture of grasslands, savanna ecosystems, and crops (0.41 ± 0.10), that C_3_ herbaceous biomass is a mixture of grasslands and crops only (0.46 ± 0.10), and that C_3_ woody biomass includes both forests and shrubland biomes (0.40 ± 0.10)^46^. We acknowledge the limitations of our approach, especially given that ${{{\rm{\tau }}}}$ and CUE may vary not only across plant functional types but also across environmental conditions, in particular temperatures^50–53^.

### Optimality model configuration and simulation

We ran the P-model on a 0.5° resolution grid at a monthly timestep over the period 1982–2016, forced by monthly mean values of daytime air temperature (*T*_air_, °C), daytime vapor pressure deficit (VPD, Pa), incident photosynthetic photon flux density (PPFD, mol m^–2^ month^–1^), the fraction of incident PPFD absorbed by foliage (*f*APAR, dimensionless), the atmospheric partial pressure of CO_2_ (*c*_a,_ Pa), root-zone volumetric soil moisture (${{{\rm{\theta }}}}$, m^3^ m^–3^) and elevation (*z*, m). Monthly mean *T*_daytime_ and VPD were calculated from minimum and maximum temperature and actual vapor pressure from the Climatic Research Unit (CRU) gridded time-series (CRU TS4.03) dataset^54^ to consider only the part of the day when photosynthesis occurs^55^. Monthly shortwave downwelling radiation (*SWdown*) was obtained from WATCH-Forcing-Data-ERA-Interim (WFDEI) data^56^ and used to calculate monthly PPFD. Supplementary Table 2 summarizes the data information and sources used in this study.

Monthly *f*APAR data were derived from the Advanced Very High Resolution Radiometer (AVHRR) Global Inventory Modeling and Mapping Studies (GIMMS) fAPAR 3 g product^57^ gridded at 0.5° resolution. Since monthly *c*_a_ varies spatially, we used the annual *c*_a_ data (μmol mol^–1^) derived from ref. ^58^ and converted them into Pa using elevations derived from WATCH-WFDEI. Monthly ${{{\rm{\theta }}}}$ (m^3^ m^–3^) over a 1 m soil depth was calculated using a modified version of the SPLASH model^59^ driven by daily precipitation, *T*_air_ and *SWdown* from the WATCH-WFDEI dataset over 1979–2018. The model ${{{\rm{\theta }}}}$ outputs were derived from ref. ^60^.

Annual data on percentage tree cover, used to estimate the fraction of C_4_ plants (see Text S1), were derived from the NASA Making Earth System Data Records for Use in Research Environments (MEaSURES) Vegetation Continuous Fields (VCF) 5KYR v001^61^ for the 1982–2016 period. The years 1994 and 2000 are missing from this dataset. We interpolated tree cover for these years by averaging values from the previous and subsequent years (1993 and 1995, and 1999 and 2001, respectively).

We used the urban areas and C_3_ and C_4_ crop distribution estimates from the LUHv2-2019 dataset (https://daac.ornl.gov/VEGETATION/guides/LUH2_GCB2019.html). Data for the remote-sensing products given at 0.05° resolution were aggregated to 0.5° resolution using the mean of all the 0.05° grid cells within the 0.5° grid cell. Climate and remote sensing data were filtered with the MODIS Land Processes Distributed Active Archive Center (LP DAAC; https://www.earthdata.nasa.gov/data/catalog/lpcloud-mcd12q1-061) snowandice and barren_sparsely_vegetated maps to retain only vegetated regions free of snow and ice.

### Optimality model evaluation and comparison

We evaluated the simulations of Δ^13^C for C_3_ and C_4_ plants using a compilation of leaf stable carbon isotope data^28^ that includes 3601 measurements for C_3_ plants and 531 measurements for C_4_ plants (see Supplementary Fig. 8a for site locations). Measured leaf δ^13^C were converted into leaf Δ^13^C using atmospheric δ^13^CO_2_ from ref. ^62^. We also evaluated the model predictions of the fraction of C_4_ plants (*F*_4_) over the 1982–2016 period using a new compilation of 2156 soil δ^13^C measurements derived from published sources specifically compiled for this study^29^ (see Supplementary Table 3 and Supplementary Fig. 8b for data information and locations). Predicted Δ^13^C values for C_3_ and C_4_ plants were converted to δ^13^C using atmospheric δ^13^CO_2_ from ref. ^62^. Since measured soil δ^13^C is an average of the stable carbon isotopic signature of soil organic matter accumulated over several years, we estimated soil δ^13^C from the model as the average of predicted δ^13^C for both C_3_ and C_4_ plants, weighted by their relative yearly fraction, over the entire period 1982–2016 to enable comparisons with the observations. We assumed that any additional isotopic fractionation within the soil is negligible over the study timeframe.

We compared the predictive skills of our approach with those from two independent, global C_4_ distribution maps. We used the map from ref. ^20^ available at https://daac.ornl.gov/cgi-bin/dsviewer.pl?ds_id=932 and the map recently developed by ref. ^12^ for the period 2001–2019 (https://zenodo.org/records/10516423)—referred to as Still2009 and Luo2024, respectively. Since these maps covered different regions of the world, we homogenized them to make them comparable. When grid points were set to “NA” but were on land (excluding deserts and snow/glacial lands), we assumed *F*_4_ = 0. As a result, the estimated *F*_4_ values derived from the maps may be slightly different from those reported in the respective publications. We predicted soil isotopic composition using each of the maps and compare them with the soil isotopic network. We also compared the mean and trends in total *F*_4_, GPP, and Δ^13^C derived from each of the maps.

We conducted additional simulations to quantify the contributions of *c*_a_, *T*_air_, VPD and *θ* to *F*_4_, GPP and Δ^13^C changes. To do so, we ran the model for four different scenarios in which we used the input of *c*_a_, *T*_air_, VPD and *θ* of the first year (1982), respectively, to simulate GPP over the whole period. We then used predicted GPP for the different scenarios to estimate *F*_4_ for each year. We calculated the difference between the original model simulations and those derived from each scenario to determine the relative contributions of *c*_a_, *T*_air_, VPD and *θ* to *F*_4_, GPP, and Δ^13^C changes.

### Simple carbon cycle model to estimate atmospheric δ^13^CO_2_

To account for differences in land ∆^13^C, biomass and carbon turnover between C_3_ and C_4_ plants, we estimated global average atmospheric δ^13^CO_2_ (‰) over 1982–2016 using the simple carbon cycle model from ref. ^30^, also used in ref. ^3^. The model simulates carbon cycling in atmospheric, oceanic, and biospheric reservoirs, and includes one atmospheric box, three biospheric boxes with different biomass and carbon turnover times, and a one-dimensional box diffusion ocean model with 43 ocean boxes. We conducted our historical simulations using the same initial model configuration and calibrated parameter ranges as in ref. ^30^. A few small changes were made to the model code: defining different Δ^13^C values for C_3_ and C_4_ plants that consider temporal changes in the fraction of C_3_ and C_4_ plants, and different carbon turnover (τ) and use efficiency (CUE) for the three biospheric boxes (see also Supplementary Note 1 for more information). We first ran the model in its standard mode, initially with a constant Δ^13^C (equal to 18‰) and then with a variable Δ^13^C based on CO_2_ changes as in ref. ^3^. We then tested the model using our global average annual model outputs for ∆^13^C and *F*_4_ by allowing box 1, with low biomass and rapid τ, to represent only C_4_ herbaceous, while boxes 2 and 3, with intermediate and high biomass and intermediate and slow τ, represent C_3_ herbaceous and woody, respectively. Since CO_2_ fertilization only impacts C_3_ photosynthesis, we assume it to be null for C_4_ photosynthesis (box 1).

## Supplementary information

Transparent Peer Review file

Supplementary Material

## Data Availability

The data that support the findings of this study are publicly available. The CRU TS4.03 datasets are available from East Anglia University (UK) at https://crudata.uea.ac.uk/cru/data/hrg/. The WATCH-WFDEI dataset is available from the International Institute for Applied Systems Analysis (Austria) via the WATCH FTP server at ftp://rfdata:forceDATA@ftp.iiasa.ac.at. The annual percentage treecover from MEaSURES VCF5KYR v001 is available at 10.5067/MEaSUREs/VCF/VCF5KYR.001. Urban areas and C_3_ and C_4_ crop distribution from LUHv2-2019 data are available at https://daac.ornl.gov/VEGETATION/guides/LUH2_GCB2019.html. The snowandice and barren_sparsely_vegetated landcover maps from MODIS LP DAAC are available at https://www.earthdata.nasa.gov/data/catalog/lpcloud-mcd12q1-061. The map of fraction of C_4_ plants from ref. ^20^ is available at 10.3334/ORNLDAAC/932. The global C_4_ distribution map developed by ref. ^12^ is available at https://zenodo.org/records/10516423. The AVHRR GIMMS fAPAR data were made available by R. Myneni (data request contact: rmyneni@bu.edu). The concentrations and isotopic compositions of atmospheric CO_2_ are available in the Supplementary Material of refs. ^30,58,62^. The soil carbon isotopic data were extracted from ref. ^29^ and is available at 10.5281/zenodo.6556096. The leaf carbon isotopic data are derived from ref. ^28^, the leaf gas-exchange data for C_4_ plants from ref. ^63^ and the share of C_4_ plants in ecosystem GPP from refs. ^64,65^, all available in the original papers. The processed climate inputs and global model outputs produced in this article are available at https://zenodo.org/records/17726762.

## Code availability

The original P-model was incorporated into Python (https://pyrealm.readthedocs.io/en/latest/), and the code modifications and additions presented in this paper are available at https://pyrealm.readthedocs.io/en/latest/users/pmodel/c3c4model.html. The Python code to run the new C_3_/C_4_ competition model, analyse simulations and create the figures is available at https://github.com/Alielav/Comms-Earth-Lavergne-et-al.-2025. The original MATLAB code to run the simple carbon cycle box model and predict atmospheric δ^13^CO_2_ is available from Heather Graven at https://github.com/heathergraven/simplemodel2020. The Python version that includes the code edits made for the purpose of this article is also available at https://github.com/Alielav/Comms-Earth-Lavergne-et-al.-2025.
