# Supplementary material for: Minimal impact of recent decline in C4 vegetation abundance on atmospheric carbon isotopic composition
Source: Commun Earth Environ. 2026 Jan 10;7(1):93. doi: 10.1038/s43247-025-03102-6 (PMC12851932; doi:10.1038/s43247-025-03102-6)
Supplement: Supplementary file 2 — Supplementary Material [file 43247_2025_3102_MOESM2_ESM.pdf]

Supplementary Material

Minimal impact of recent decline in C₄ vegetation abundance on atmospheric carbon isotopic composition

Supplementary Note 1

The P model for C₃ and C₄ plants

The P model¹⁻³ is an optimality-based light-use efficiency model which simulates ecosystem GPP as a function of air temperature (T_{air}), vapour pressure deficit (VPD), incident photosynthetic photon flux density (PPFD), the fraction of incident PPFD absorbed by foliage (f_{APAR}), the atmospheric partial pressure of CO₂ (c_a), volumetric soil moisture (θ) and atmospheric pressure via elevation (z) on time steps of a day to a month. Accumulated GPP is given by:

$$GPP = \phi_0(T) I_{\text{abs}} m_j \sqrt{1 - (c^*/m_j)^{2/3}} \quad (1a)$$

where

$$m_j = \frac{c_i - \Gamma^*}{c_i + 2\Gamma^*} \quad (1b)$$

$$c_i = (c_a - \Gamma^*) \frac{\xi}{\xi + \sqrt{VPD}} + \Gamma^* \quad (1c)$$

$$\xi = \sqrt{\left(\beta \frac{(K + \Gamma^*)}{1.6 \eta^*} \right)} \quad (1d)$$

ϕ_0 is the temperature-dependent intrinsic quantum yield (mol CO₂ mol⁻¹) which differs for C₃ and C₄ plants⁴. I_{abs} is the PPFD intercepted and absorbed by the canopy (mol m⁻² s⁻¹). c_i is the leaf internal partial pressure of CO₂ (Pa). Γ^* is the photorespiratory compensation point (Pa), which depends on temperature and atmospheric pressure. η^* is the viscosity of water, relative to its value at 25°C (dimensionless). K is the effective Michaelis-Menten coefficient of Rubisco (Pa). c^* is a dimensionless constant equal to 0.41 which has been estimated from observed ratios of Rubisco and electron-transport photosynthetic capacities. β (unitless) is the ratio of cost factors for carboxylation and transpiration at 25°C.

ϕ_0 , Γ^* and β values vary between C₃ and C₄ photosynthesis. The temperature-dependent ϕ_0 for C₃ and C₄ plants are calculated as in [4]. As photorespiration is strongly reduced, Γ^* is assumed to be zero for C₄ plants. Since β varies with changes in plant-available soil water for C₃ plants, the function calibrated using stable carbon isotopic measurements from leaves and tree rings⁵ was used to calculate β as:

$$\beta_{C3} = \exp^{1.73\theta+4.55} \quad (2)$$

where θ is the soil moisture content ($\text{m}^3 \text{m}^{-3}$) in the first metre of soil.

An empirical analysis using more than 1100 C_4 leaf gas exchange measurements in [6] showed that β in C_4 plants does not significantly depend on θ (Supplementary Figure 9). We therefore used a fixed value of 35, which is the median β value from the regression.

P model predictions of GPP have been extensively validated against eddy-covariance flux data in other studies². [4] showed that it can reproduce positive and negative trends in GPP at flux sites with long records.

The model of C_3/C_4 competition

A quantity representing the “advantage” of C_4 plants (Adv_4) is calculated as follows:

$$Adv_4 = \frac{\sum GPP_4 - \sum GPP_3}{\sum GPP_3} \quad (3)$$

where GPP_3 and GPP_4 are monthly potential GPP values for C_3 and C_4 plants, respectively, both summed over 12 months.

The share of C_4 plants in the total GPP (Sh_4) is then estimated by fitting a general logistic curve to the simulated Adv_4 and observed share of C_4 plants in ecosystem GPP, using data from [7,8]:

$$Sh_4 = \{1 + \exp^{-k(Adv_4 - q)}\}^{-1} \quad (4)$$

where q is the value of Adv_4 that yields $Sh_4 = 0.5$, and k is the steepness of the curve at that point. Non-linear least squares regression (Supplementary Figure 10) gave values of $k = 4.26$ and $q = 0.23$ ($R^2 = 0.29$ and $RMSE = 0.271$). The uncertainties in predicted Adv_4 and Sh_4 could contribute to uncertainties in the predictions of the fraction of C_4 plants. However, the lack of additional Sh_4 data in the literature for the observed contribution of C_4 plants to total ecosystem GPP prevents us refining this relationship. Despite this limitation, our logistic prediction shows that Sh_4 increases with Adv_4 , which is what is expected from the theory.

When water and nutrients are not limiting, C_3 trees outcompete C_4 grasses through shading even where the C_4 pathway would yield higher rates of photosynthesis⁹. To exclude C_4 plants in this situation, we defined an additional function relating the proportion of GPP from trees to total potential GPP based on a power function for the relationship between prescribed annual mean percentage tree cover (TC) and the simulated annual GPP of C_3 plants ($\sum GPP_3$, $\text{kgC m}^{-2} \text{yr}^{-1}$) for grid cells with $Sh_4 > 0.35$ ($R^2 = 0.74$ and $RMSE = 16.87$).

$$h = \frac{TC(\sum GPP_3)}{TC(cutoff)} = \frac{14.85 (\sum GPP_3)^{1.46} - 7.17}{15.85 (cutoff)^{1.46} - 7.17} \quad (5)$$

where h is the predicted proportion of GPP from trees ($h \leq 1$), and $cutoff$ is the annual GPP_3 values determining the limits of closed forest.

We estimated the cut-off point to be where $GPP_{C3} > 2.8 \pm 0.2 \text{ kgC m}^{-2} \text{ yr}^{-1}$ (approximately $58 \pm 2\%$ of TC) based on the bimodal distribution between forest and non-forest grid cells shown in the density plots of GPP_{C3} and TC (Supplementary Figure 11). We used this cut-off to delimit grid cells where GPP is from C_3 trees, whatever the Sh_4 value. h is then removed to Sh_4 to determine the potential share of C_4 in GPP:

$$Sh_{4,pot} = Sh_4 (1 - h) \quad (6)$$

[10] showed experimentally that C_4 plants cannot grow below -20 to -24°C . We therefore set $Sh_{4,pot}$ to 0 in areas where the mean T_{air} of the coldest month is $< -24^\circ\text{C}$. We then converted $Sh_{4,pot}$ to fraction of C_4 plants ($F_{4,pot}$) using the emergent constraint from [11] calculated as the share of C_4 plants divided by 1.13.

Since the total fractions of C_3 and C_4 plants consist of natural ecosystems (trees, grasses) and crops, we estimated the C_4 fraction of natural ecosystems ($F_{4,nat}$) by removing the fraction of human managed areas (C_3 and C_4 crops and urban areas extracted from LUHv2-2019 data; noted $F_{3,crops}$, $F_{4,crops}$ and F_{urban} , respectively) from $F_{4,pot}$ as:

$$F_{4,nat} = F_{4,pot} - (F_{3,crops} + F_{4,crops}) - F_{urban} \quad (7)$$

We then calculated the total fraction of C_4 and C_3 plants ($F_{4,tot}$ and $F_{3,tot}$) considering natural ecosystems ($F_{4,nat}$ and $F_{3,nat}$) and crops ($F_{4,crops}$ and $F_{3,crops}$) as:

$$F_{4,tot} = F_{4,nat} + F_{4,crops} \quad (8a)$$

$$F_{3,tot} = 1 - F_{4,tot} = F_{3,nat} + F_{3,crops} \quad (8b)$$

We estimated C_3 , C_4 and total ($C_3 + C_4$) GPP from their respective potential GPP as:

$$GPP_{C3} = GPP_{C3,pot} F_{3,tot} \quad (9a)$$

$$GPP_{C4} = GPP_{C4,pot} F_{4,tot} \quad (9b)$$

$$GPP_{tot} = GPP_{C3} + GPP_{C4} \quad (9c)$$

We aggregated GPP for C_3 , C_4 and all ($C_3 + C_4$) plants from each grid cell weighted by grid-cell area for each prediction (in PgC yr^{-1}).

Carbon isotopic discrimination calculation

The land stable carbon isotopic discrimination ($\Delta^{13}\text{C}$) is calculated as:

$$\Delta^{13}\text{C} = \frac{f_{4,herb} \Delta_4 \tau_{4,herb} + f_{3,herb} \Delta_3 \tau_{3,herb} + f_{3,woody} \Delta_3 \tau_{3,woody}}{\tau_{4,herb} + \tau_{3,herb} + \tau_{3,woody}} \quad (10)$$

where Δ_4 and Δ_3 are the carbon isotope discrimination in C_4 and C_3 plants, respectively. $\tau_{4,herb}$, $\tau_{3,herb}$, $\tau_{3,woody}$ are the carbon turnover times and $f_{4,herb}$, $f_{3,herb}$ and $f_{3,woody}$ are the fractions of C_4 herbaceous, C_3 herbaceous and C_3 woody plants, respectively, adjusted to account for lower impact of C_3/C_4 fraction on $\Delta^{13}\text{C}$ than on GPP.

Δ_3 was calculated, following the standard formulation¹², as follows:

$$\Delta_3 = a_s + (b - a_s)\chi_3 - f \frac{r^*}{c_a} \quad (11)$$

with $a_s = 4.4\text{‰}$, $b = 28 \pm 2\text{‰}$, $f = 12 \pm 4\text{‰}$ and χ_3 is the predicted ratio of c_i to c_a for C_3 plants.

There is considerable uncertainty about which discrimination model to use for C_4 plants¹³, we therefore calculated Δ_4 from a linear regression between observed Δ_4 derived from 531 leaf $\Delta^{13}\text{C}$ measurements compiled by [14] and the predicted ratio of c_i to c_a for C_4 plants, χ_4 (Supplementary Figure 12) as follows:

$$\Delta_4 = 13.95 - 17.04 \chi_4 \quad (12)$$

Δ_3 and Δ_4 are calculated for each month (*month*) and weighted by each month's C_3 and C_4 GPP to obtain annual averages of Δ_3 and Δ_4 :

$$\Delta_{x,yr} = \sum_t \frac{\Delta_{x,month} \times \text{GPP}_{x,month}}{\sum_t \text{GPP}_{x,month}} \quad (13)$$

with x being the plant type (C_3 or C_4) selected.

$f_{4,tot}$ and $f_{3,tot}$ are calculated as follow:

$$f_{4,herb} = F_{4,tot,t0} - (F_{4,tot,t0} - F_{4,tot}) \text{CUE}_{4,herb} \quad (14a)$$

$$f_{3,herb} = F_{3,tot,t0} - (F_{3,tot,t0} - F_{3,tot}) \text{CUE}_{3,herb} \quad (14b)$$

$$f_{3,woody} = F_{3,tot,t0} - (F_{3,tot,t0} - F_{3,tot}) \text{CUE}_{3,woody} \quad (14c)$$

$F_{4,tot,t0}$ and $F_{3,tot,t0}$ are the fractions of C_4 and C_3 plants, respectively, at time $t0$ - the first year of the records (1982). $\text{CUE}_{4,herb}$, $\text{CUE}_{3,herb}$ and $\text{CUE}_{3,woody}$ are the carbon use efficiencies (CUE,

unitless; or ratio of net to gross primary productivity, NPP/GPP) for C₄ herbaceous, C₃ herbaceous and C₃ woody plants, respectively.

Atmospheric $\delta^{13}\text{C}$ simulations

We ran the simple carbon cycle model from [15] to predict atmospheric $\delta^{13}\text{C}$ over the recent decades. We used the same model configuration and input data, and parameter ranges as in the study, but made a few edits in the code. We defined different $\Delta^{13}\text{C}$ values for C₃ and C₄ plants and consider temporal changes in the fraction of C₃ and C₄ plants.

Historical atmospheric CO₂ concentration was specified by global annual mean data from [16], which is almost identical as that from [17] for the studied period. Historical fossil fuel CO₂ emissions data were given by the Community Emissions Data System (CEDS)¹⁸. Historical land use CO₂ emissions data were given by the Coupled Climate Carbon Cycle Model Intercomparison Project (C4MIP)¹⁹. The $\delta^{13}\text{C}$ of fossil fuel emissions was specified by [20] for 1850 to 2013 and then kept fixed at the 2013 value, -27.73‰, for the period 2013 to 2016.

Simulations for 1850-1981 period were conducted following a spin-up of 11850 years. Atmospheric $\delta^{13}\text{C}$ was specified by the historical data from [21] for 1850-1981 and then atmospheric $\delta^{13}\text{C}$ were simulated prognostically for 1982-2016. In the simulations with original model configuration, a sensitivity of $\Delta^{13}\text{C}$ to atmospheric CO₂ concentration was included following [22] and [23]. The $\Delta^{13}\text{C}$ formulation in equations 3 and 4 from [22] was used, beginning from $\Delta^{13}\text{C}$ of 17‰ in 1850. In the simulations with a modified version of the carbon cycle model, the global annual model outputs of $\Delta^{13}\text{C}$ for C₃ and C₄ plants ($\Delta_{yr,3}$ and $\Delta_{yr,4}$) and fractions of C₃ and C₄ plants ($F_{3,tot}$ and $F_{4,tot}$) were used.

Supplementary Note S2

Sensitivity of predicted GPP to environmental drivers for C₃ and C₄ photosynthesis.

We performed a sensitivity analysis of the response of predicted GPP to individual environmental drivers for C₃ and C₄ plants to examine the differences between the two photosynthetic pathways. We used standard values to run the model ($T_{\text{air}} = 25^\circ\text{C}$, VPD = 0.80 kPa, PPFD = 800 mol m⁻² month⁻¹, $f\text{APAR} = 0.90$, $c_a = 400 \mu\text{mol mol}^{-1}$, $\theta = 0.4 \text{ m}^3 \text{ m}^{-3}$ and $P_{\text{atm}} = 101.325 \text{ kPa}$) and changed one variable at a time over the range of its potential values

(Supplementary Figure 6). We also estimated the relative effects of the environmental drivers of C₃ and C₄ plants of predicted GPP over 1982-2016 under real conditions using a generalized least square model (Supplementary Figure 7). We first standardized the predictors values by subtracting the mean and then dividing by the standard deviation.

The sensitivity of the two photosynthetic pathways to increasing photosynthetically active radiation (the product of PPFD and fAPAR) are similar (Supplementary Figures 6c-d and 7a-b). This is because, on the one hand, the light use efficiency is reduced as CO₂ is subsaturating for C₃ plants while, on the other hand, ϕ_0 is lower for C₄ plants²⁴ as the CO₂ concentrating mechanism has an energetic cost²⁵. Increased T_{air} tends to favour C₄ photosynthesis because C₄ plants can reduce CO₂ losses by suppressing photorespiration^{26,27}, while it decreases C₃ photosynthesis mostly from average growing season temperatures with photorespiration (Supplementary Figures 6a and 7). While an increase in c_a stimulates C₃ photosynthesis²⁸, it does not have a significant impact on C₄ photosynthesis (Supplementary Figures 6e and 7a-b). The model predicts the T_{air} “crossover” at which the quantum yield of C₃ plants equals that of C₄ plants, above which C₄ plants can fix carbon at a faster rate than C₃ plants^{9,29,30}; and also the CO₂ “crossover”, above which C₃ plants can fix carbon at a faster rate than C₄ plants. These thresholds of T_{air} and c_a vary with changing environmental conditions, increasing with an increase in c_a and in T_{air} , respectively³¹. Increasing atmospheric aridity (vapour pressure deficit, VPD) decreases modelled GPP for C₃ plants but not C₄ plants, consistent with experimental studies³² (Supplementary Figures 6e and 7a-b). Thus, the current increase in c_a benefits C₃ plants³³, while the associated increases in T_{air} and VPD might provide a countervailing advantage to C₄ plants³⁴. The success of C₃/C₄ competition in the model is therefore determined by the distinct effects of c_a , T_{air} and VPD on C₃ and C₄ photosynthesis.

Sensitivity analysis of predicted $\Delta^{13}\text{C}$ F_4 to environmental drivers

Similarly to GPP, we estimated the relative effects of elevated c_a , T_{air} and water stress as indicated by high VPD and low θ on $\Delta^{13}\text{C}$ using a generalized least square model over 1982-2016. We first standardized the predictors values by subtracting the mean and then dividing by the standard deviation.

$\Delta^{13}\text{C}_{\text{C3}}$ increases with higher T_{air} , c_a and θ but decreases with high VPD and low atmospheric pressure as indicated by high elevation z (Supplementary Figure 7c). $\Delta^{13}\text{C}_{\text{C4}}$ responds inversely to these environmental drivers (Supplementary Figure 7d). These patterns of variations were expected as both $\Delta^{13}\text{C}_{\text{C3}}$ and $\Delta^{13}\text{C}_{\text{C4}}$ depend on the ratio of leaf intercellular to ambient CO_2 (c_i/c_a) and mainly respond to the environmental drivers of c_i/c_a . While $\Delta^{13}\text{C}_{\text{C3}}$ tends to increase with c_i/c_a ¹², $\Delta^{13}\text{C}_{\text{C4}}$ decreases with c_i/c_a ^{13,35} (see also Supplementary Figure 12).

Supplementary Table 1 Calibrated parameter sets used for the carbon cycle box model¹⁵. Eddy diffusivity (Keddy, m²y⁻¹), atmospheric CO₂ residence time with respect to exchange with the mixed layer of 9–11 year (τ_{am}), CO₂ fertilization factor (β , unitless), atmospheric CO₂ residence time with respect to biospheric exchange (τ_{ab}), biospheric residence time or carbon turnover (τ_{ba}) for box 1 to 3, respectively.

Keddy<br>(m ² y ⁻¹)	τ_{am}<br>(year)	β	τ_{ab1}<br>(year)	τ_{ab2}<br>(year)	τ_{ab3}<br>(year)	τ_{ba1}<br>(year)	τ_{ba2}<br>(year)	τ_{ba3}<br>(year)
4000	10	0.4	27.1	21.6	210.9	2.4	24.8	580.2
4000	10	0.4	33.8	25.6	61.9	2	13.4	212.8
4000	11	0.4	21.2	31.5	53	2.6	24.1	165.7
3000	9	0.4	33.8	25.6	61.9	2	13.4	212.8
3000	10	0.4	33.8	25.6	61.9	2	13.4	212.8
3000	11	0.4	33.8	25.6	61.9	2	13.4	212.8
3000	11	0.4	21.2	31.5	53	2.6	24.1	165.7
4000	9	0.4	38.1	23	107.8	2	15.7	353.2
3000	9	0.4	38.1	23	107.8	2	15.7	353.2
5000	11	0.4	23.2	24.4	105.4	2.5	26.2	299.4
4000	11	0.4	23.2	24.4	105.4	2.5	26.2	299.4
3000	10	0.4	23.2	24.4	105.4	2.5	26.2	299.4
3000	11	0.4	23.2	24.4	105.4	2.5	26.2	299.4
3000	9	0.4	27.1	21.6	210.9	2.4	24.8	580.2
3000	10	0.4	27.1	21.6	210.9	2.4	24.8	580.2
3000	11	0.4	21.7	20.1	232.6	2.6	34.7	502.4

209 **Supplementary Table 2** Climate and satellite remote sensing input data used in this study

210

Dataset	Spatial resolution	Temporal resolution	Duration	Source
min and max temperatures, actual vapor pressure to calculate T_{daytime} and VPD	0.5°x0.5°	monthly	1901-2018	CRU TS-4.03<br>https://crudata.uea.ac.uk/cru/data/hrg/
precipitation, T_{air} , SW_{down} to calculate PPFD and θ	0.5°x0.5°	daily	1979-2018	WATCH-WFDEI<br>ftp://rfddata:forceDATA@ftp.iiasa.ac.at
annual % treecover	0.05°x0.05°	yearly	1982-2016	MEaSURES VCF5KYR v001<br>https://doi.org/10.5067/MEaSURES/VCF/VCF5KYR.001
fAPAR	0.05°x0.05°	daily	1982-2016	AVHRR GIMMS fAPAR 3g product -<br>https://www.ncei.noaa.gov/products/climate-data-records/leaf-area-index-and-fapar updated by R. Myneni
urban areas - C_3 and C_4 crop distribution	0.05°x0.05°	yearly	850-2019	LUHv2-2019 dataset<br>https://daac.ornl.gov/VEGETATION/guides/LUH2_GCB2019.html
snowandice, barren_sparsely_vegetated	0.05°x0.05°	yearly	2001-2022	MODIS LP DAAC<br>https://www.earthdata.nasa.gov/data/catalog/lpclo-ud-mcd12q1-061
atmospheric CO ₂ concentration	global	yearly	1750-2015	^{16,17}
$\delta^{13}\text{C}$ of atmospheric CO ₂	global	yearly	1850-2015	^{15,21}
fossil fuel CO ₂ emissions	global	yearly	1750–2014	Community Emissions Data System ¹⁸
land use CO ₂ emissions	0.5° × 0.5°	yearly	1850–2014	C4MIP ¹⁹<br>(CMIP6_C4MIP_landuse_emissions.nc)<br>http://c4mip.net/index.php?id=3455
$\delta^{13}\text{C}$ of fossil fuel emissions	global	yearly	1850-2013	²⁰

211

212 **Supplementary Table 3** Soil isotopic data location and references selected from Dong et al.
 213 (2022)³⁶ compilation. All data have been previously published and made available in the
 214 original publications.

215

Author	Region	References	Data access
Accoe et al. (2003)	Belgium	Accoe et al. (2003), Relationship between soil organic C degradability and the evolution of the $\delta^{13}\text{C}$ signature in profiles under permanent grassland. <i>Rapid Commun. Mass Spectrom.</i> , 17: 2591-2596.	³⁷
Acton et al. (2013)	USA<br>Australia	Acton et al. (2013), Carbon isotopes for estimating soil decomposition and physical mixing in well-drained forest soils, <i>J. Geophys. Res. Biogeosci.</i> , 118, 1532– 1545.	³⁷
Amundson et al. (1994)	USA	Amundson et al. (1994), The relationship of precipitation seasonality to the flora and stable isotope chemistry of soils in the Vizcaino desert, Baja California, Mexico, <i>J. Arid Environ.</i> , 28(4), 265-279.	³⁷
Andreeva et al. (2013)	Mongolia	Andreeva et al. (2013), Stable isotope ($\delta^{13}\text{C}$, $\delta^{15}\text{N}$, $\delta^{18}\text{O}$) record of soils in Buryatia, southern Siberia: Implications for biogeochemical and paleoclimatic interpretations, <i>Quaternary International</i> 290-291 (2013) 82-94.	³⁷
Arai & Tokuchi (2010)	Japan	Arai & Tokuchi (2010), Soil organic carbon accumulation following afforestation in a Japanese coniferous plantation based on particle-size fractionation and stable isotope analysis, <i>Geoderma</i> 159 (2010) 425–430.	³⁷
Aranibar & Macko (2005)	Southern Africa	Aranibar & Macko (2005), SAFARI 2000 Plant and Soil C and N Isotopes, Southern Africa, 1995-2000. ORNL DAAC, Oak Ridge, Tennessee, USA. https://doi.org/10.3334/ORNLDAAAC/783	³⁸
Baisden et al. (2002)	USA	Baisden et al. (2002) A multi-isotope C and N modeling analysis of soil organic matter turnover and transport as a function of soil depth in a California annual grassland soil chronosequence, <i>Global Biogeochem. Cycles</i> , 16, 1135.	³⁷
Balesdent et al. (1993)	France	Balesdent et al. (1993), Site-Related ^{13}C of Tree Leaves and Soil Organic Matter in a Temperate Forest. <i>Ecology</i> , 74: 1713-1721.	³⁷
Becker-Heidmann & Scharpenseel (1986)	Germany	Becker-Heidmann & Scharpenseel (1986), Thin Layer $\delta^{13}\text{C}$ and D^{14}C Monitoring of “Lessive” Soil Profiles. <i>Radiocarbon</i> , 28(2A), 383-390.	³⁷
Becker-Heidmann & Scharpenseel (1989)	Germany	Becker-Heidmann & Scharpenseel (1989), Carbon Isotope Dynamics in Some Tropical Soils. <i>Radiocarbon</i> , 31(3), 672-679.	³⁷

Bostrom et al. (2007)	Sweden	Boström et al. (2007), Isotope fractionation and ^{13}C enrichment in soil profiles during the decomposition of soil organic matter. <i>Oecologia</i> 153, 89–98.	37
Camino-Serrano et al. (2019)	France	Camino-Serrano et al. (2019), Including stable carbon isotopes to evaluate the dynamics of soil carbon in the land surface model ORCHIDEE. <i>Journal of Advances in Modeling Earth Systems</i> , 11, 3650–3669.	39
Campbell et al. (2009)	USA	Campbell et al. (2009), Carbon and nitrogen isotopic measurements from southern Appalachian soils: Assessing soil carbon sequestration under climate and land-use variation, <i>J. Environ. Eng.</i> , 135, 439–448.	37
Chen et al. (2005)	China	Chen et al. (2005), Spatial and temporal distribution of carbon isotopes in soil organic matter at the Dinghushan Biosphere Reserve, South China. <i>Plant Soil</i> 273, 115–128.	37
Chiang et al. (2004)	China	Chiang et al. (2004), Change in the grassland-forest boundary at Ta-Ta-Chia long term ecological research (LTER) site detected by stable isotope ratio of soil organic matter. <i>Chemosphere</i> , 54: 217–224.	37
Desjardins et al. (1994)	Brazil/Cameroun	Desjardins et al. (1994). Organic carbon and ^{13}C contents in soils and soil size-fractions, and their changes due to deforestation and pasture installation in eastern Amazonia. <i>Geoderma</i> 61:103–118.	37
Diochon & Kellman (2008)	Canada	Diochon & Kellman (2008), Natural abundance measurements of ^{13}C indicate increased deep soil carbon mineralization after forest disturbance, <i>Geophys. Res. Lett.</i> , 35.	37
Ehleringer et al. (2010)	Brazil	Ehleringer et al. (2010), LBA-ECO CD-02 Carbon, Nitrogen, Oxygen Stable Isotopes in Organic Material, Brazil. ORNL DAAC, Oak Ridge, Tennessee, USA. https://doi.org/10.3334/ORNLDAAAC/983	40
Feng et al. (1999)	USA	Feng et al. (1999), Distribution, accumulation and fluxes of soil carbon in four monoculture lysimeters at San Dimas Experimental Forest, California, <i>Geochim. Cosmochim. Acta</i> , 63(9), 1319-1333.	37
Garten & Hanson (2006)	USA	Garten & Hanson (2006), Measured forest soil C stocks and estimated turnover times along an elevation gradient. <i>Geoderma</i> 136:342–352.	37
Garten et al. (2000)	USA	Garten et al. (2000), Climate controls on forest soil C isotope ratios in the Southern Appalachian mountains. <i>Ecology</i> , 81: 1108-1119.	37
Gouveia et al. (2002)	Brazil	Gouveia et al. (2002), Carbon isotopes in charcoal and soils in studies of paleovegetation and climate changes during the late Pleistocene and the Holocene in the southeast and centerwest regions of Brazil, <i>Global and Planetary Change</i> 33: 95 – 106.	37
Gregorich et al. (1995)	Canada	Gregorich et al. (1995) Turnover of soil organic matter and storage of corn residue carbon estimated from natural ^{13}C abundance. <i>Canadian Journal of Soil Science</i> . 75(2): 161-167.	37

Guillaume et al. (2015)	Indonesia	Guillaume et al. (2015), Losses of soil carbon by converting tropical forest to plantations: erosion and decomposition estimated by $\delta^{13}\text{C}$. <i>Glob Change Biol</i> , 21: 3548-3560.	37
Jin et al. (2014)	China	Jin et al. (2014), Natural vegetation restoration is more beneficial to soil surface organic and inorganic carbon sequestration than tree plantation on the Loess Plateau of China, <i>Sci. Total Environ.</i> , 485, 615-623.	37
Koarashi et al. (2005)	Japan	Koarashi et al. (2005), Radiocarbon and stable carbon isotope compositions of chemically fractionated soil organic matter in a temperate-zone forest, <i>J. environ. Radioactiv.</i> , 79(2), 137-156.	37
Krull et al. (2002)	Kenya	Krull et al. (2002), Soil organic matter decomposition and turnover in a tropical Ultisol: evidence from $\delta^{13}\text{C}$, $\delta^{15}\text{N}$ and geochemistry, <i>Radiocarbon</i> , 44(1), 93-112.	37
Qi (2017)	Tibet, China	Qi (2017): Distribution of Organic Carbon Isotope Composition for Modern Soils from the Eastern Margin of the Tibetan Plateau and its Main Controlling Factors, Doctor, China University of Geosciences, Wuhan.	41
Lu et al. (2004)	Tibet, China	Lu et al. (2004): Distribution of carbon isotope composition of modern soils on the Qinghai-Tibetan Plateau, <i>Biogeochemistry</i> , 70, 273- 297.	41
Laskar et al. (2016)	India	Laskar et al. (2016), Stable and radioactive carbon in forest soils of Chhattisgarh, Central India: Implications for tropical soil carbon dynamics and stable carbon isotope evolution. <i>Journal of Asian Earth Sciences</i> 123, 47-57.	37
Lawrence et al. (2015)	USA	Lawrence et al. (2015), Long-term controls on soil organic carbon with depth and time: A case study from the Cowlitz River Chronosequence, WA USA. <i>Geoderma</i> 247-248, 73-87.	37
Mariotti & Peterschmitt (1994)	India	Mariotti & Peterschmitt (1994), Forest savanna ecotone dynamics in India as revealed by carbon isotope ratios of soil organic matter, <i>Oecologia</i> , 97(4), 475-480.	37
Martin et al. (1990)	Ivory Coast	Martin et al. (1990), Estimate of organic matter turnover rate in a savanna soil by ^{13}C natural abundance measurements, <i>Soil Biol. Biochem.</i> , 22(4), 517-523.	37
Marty et al. (2011)	Canada	Marty et al. (2011), Isotopic compositions of S, N and C in soils and vegetation of three forest types in Québec, Canada, <i>Appl. Geochem.</i> , 26(12), 2181-2190.	37
Masiello et al. (2004)	USA	Masiello et al. (2004), Weathering controls on mechanisms of carbon storage in grassland soils, <i>Global Biogeochemical Cy.</i> , 18(4), GB4023.	37
Natehoffer & Fry (1988)	USA	Natehoffer & Fry (1988), Controls on natural nitrogen-15 and carbon-13 abundances in forest soil organic matter, <i>Soil Sci. Soc. Am. J.</i> , 52(6), 1633-1640.	37

Nel et al. (2018)	World	Nel et al. (2018), Correspondence between $\delta^{13}\text{C}$ and $\delta^{15}\text{N}$ in soils suggests coordinated fractionation processes for soil C and N. <i>Plant Soil</i> 423, 257–271.	42
Peri et al. (2012)	Patagonia	Peri et al. (2012), Carbon ($\delta^{13}\text{C}$) and nitrogen ($\delta^{15}\text{N}$) stable isotope composition in plant and soil in Southern Patagonia's native forests. <i>Glob. Change Biol.</i> , 18: 311-321.	43
Pessenda et al. (1998)	Brazil	Pessenda et al. (1998), Super ^{14}C dating and stable carbon isotopes of soil organic matter in forest-savanna boundary areas in the southern Brazilian Amazon region. <i>Radiocarbon</i> , 44(1), 93-112.	37
Prest et al. (2014)	Canada	Prest et al. (2014), Mineral soil carbon and nitrogen still low three decades following clearcut harvesting in a typical Acadian Forest stand, <i>Geoderma</i> , 214, 62-69.	37
Schneckenberger & Kuzyakov (2007)	Germany	Schneckenberger & Kuzyakov (2007), Carbon sequestration under <i>Miscanthus</i> in sandy and loamy soils estimated by natural ^{13}C abundance, <i>J. Plant Nutr. Soil Sc.</i> , 170(4), 538-542.	37
Schwendenmann & Pendall (2006)	Panama	Schwendenmann & Pendall (2006), Effects of forest conversion into grassland on soil aggregate structure and carbon storage in Panama: evidence from soil carbon fractionation and stable isotopes, <i>Plant and Soil</i> , 288(1-2), 217-232.	37
Sommer et al. 2012	Australia	Sommer et al. (2012), Limited carbon and mineral nutrient gain from mycorrhizal fungi by adult Australian orchids. <i>American Journal of Botany</i> , 99: 1133-1145.	44
Tedersoo et al. (2012)	Gabon	Tedersoo et al. (2012), Enzymatic activities and stable isotope patterns of ectomycorrhizal fungi in relation to phylogeny and exploration types in an afro-tropical rain forest. <i>New Phytologist</i> , 195: 832-843.	45
Teger & Dorr (1996)	Germany	Teger & Dorr (1996). ^{14}C Measurements of Soil Organic Matter, Soil CO_2 and Dissolved Organic Carbon (1987–1992). <i>Radiocarbon</i> , 38(2), 247-251.	37
Telles et al. (2003)	Brazil	Telles et al. (2003), Influence of soil texture on carbon dynamics and storage potential in tropical forest soils of Amazonia, <i>Global Biogeochemical Cy.</i> , 17(2). 1040.	37
Torn et al. (2002)	Russia	Torn et al. (2002), Organic carbon and carbon isotopes in modern and 100 year old soil archives of the Russian steppe, <i>Glob. Change Biol.</i> , 8(10), 941-953.	37
Tu et al. (2011)	China	Tu et al. (2011), Sources of dissolved organic carbon in forest soils: evidences from the differences of organic carbon concentration and isotope composition studies. <i>Environmental Earth Sciences</i> 63, 723-730.	37
Wang et al. (2017)	Mongolia, China	Wang et al. (2017) Depth profiles of soil carbon isotopes along a semi-arid grassland transect in northern China. <i>Plant Soil</i> 417, 43–52.	37

Wynn et al. (2005)	Australia	Wynn et al. (2005), Rayleigh distillation and the depth profile of $^{13}\text{C}/^{12}\text{C}$ ratios of soil organic carbon from soils of disparate texture in Iron Range National Park, Far North Queensland, Australia, <i>Geochim. cosmochim. Ac.</i> , 69(8), 1961-1973.	37
Wynn et al. (2006)	USA	Wynn et al. (2006), Stable carbon isotope depth profiles and soil organic carbon dynamics in the lower Mississippi Basin, <i>Geoderma</i> , 131(1), 89-109.	37
Yonekura et al. (2012)	Indonesia	Yonekura et al. (2012), Dynamics of soil carbon following destruction of tropical rainforest and the subsequent establishment of Imperata grassland in Indonesian Borneo using stable carbon isotopes, <i>Glob. Change Biol.</i> , 18(8), 2606-2616.	37
Zummo & Friedland (2011)	USA	Zummo & Friedland (2011), Soil carbon release along a gradient of physical disturbance in a harvested northern hardwood forest, <i>Forest Ecol. Manag.</i> , 261(6), 1016-1026.	37

Supplementary Figure 1 Workflow to estimate fraction of natural C₄ plants from the optimality P model and observations. The green box indicates the P model, the blue boxes are for the observations and the yellow box indicates the emergent constraint.

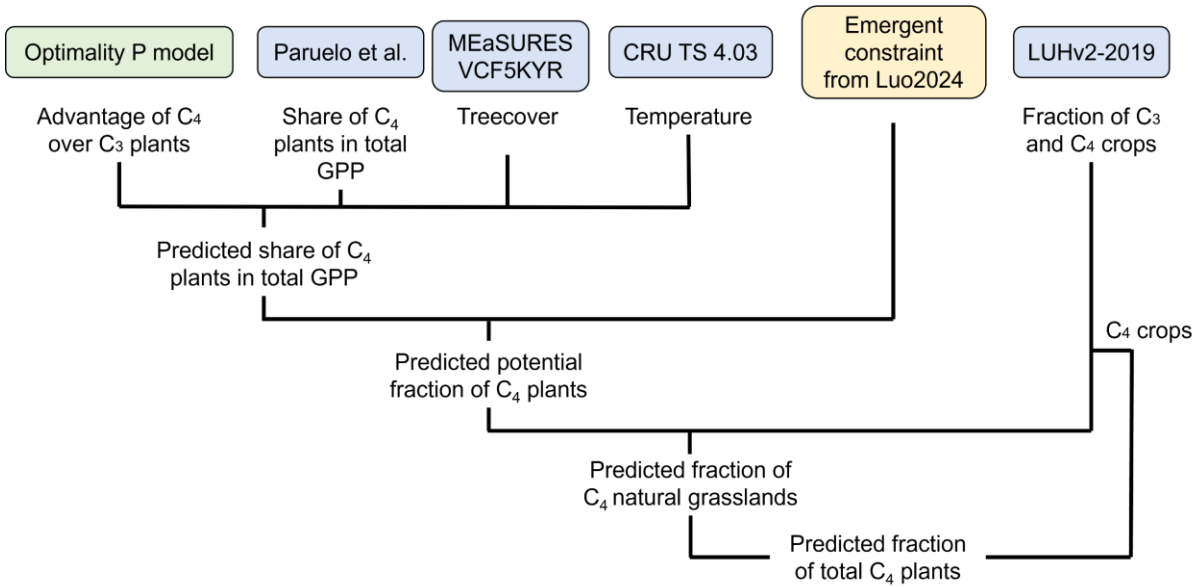

Supplementary Figure 2 Temporal trend in the global gross primary production (GPP, in $\text{gC m}^{-2} \text{yr}^{-2}$). (a) C_3 plants, (b) C_4 plants and (c) all types of plants.

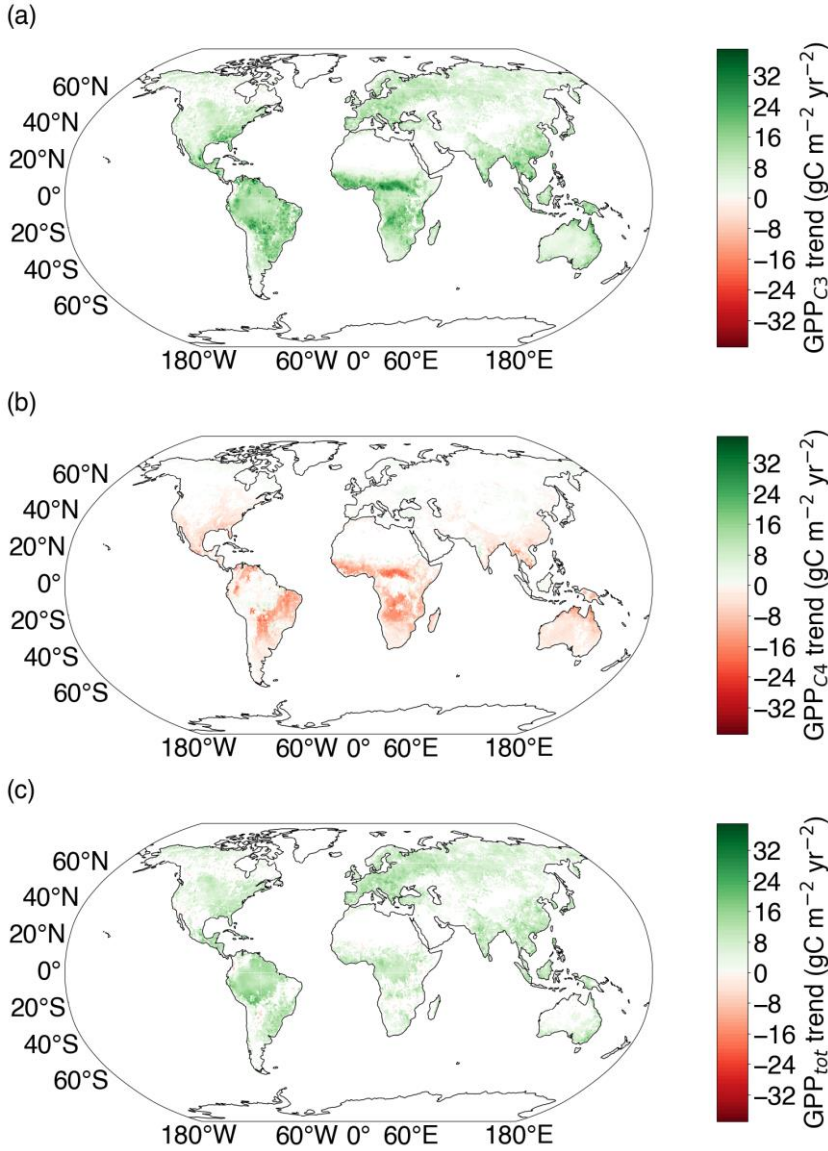

Supplementary Figure 3 Temporal trend in the carbon isotopic discrimination ($\Delta^{13}\text{C}$, in ‰ yr^{-1}). (a) C_3 plants, (b) C_4 plants and (c) all types of plants.

(a)

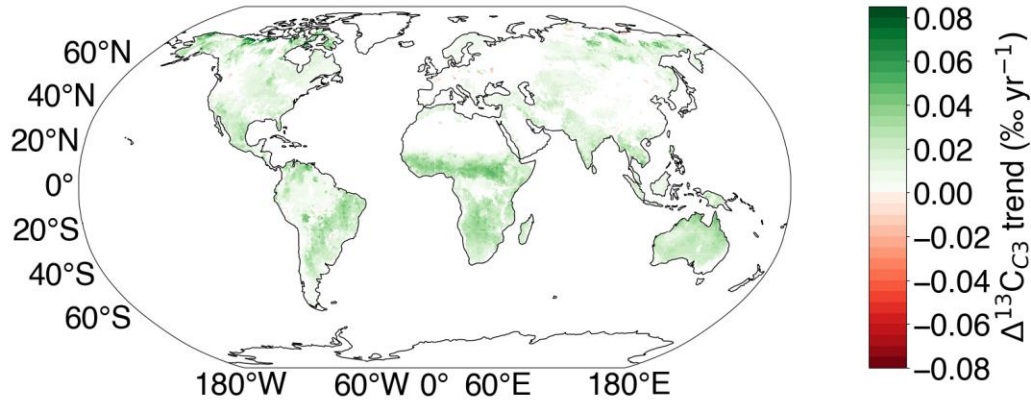

(b)

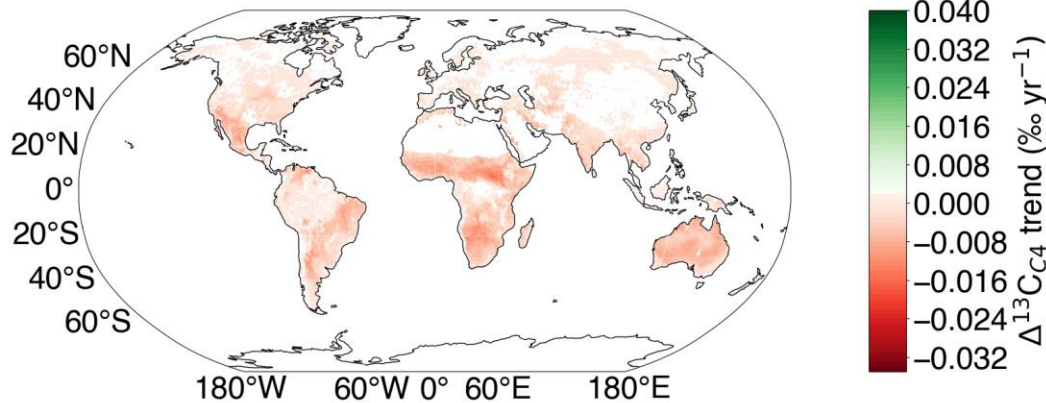

(c)

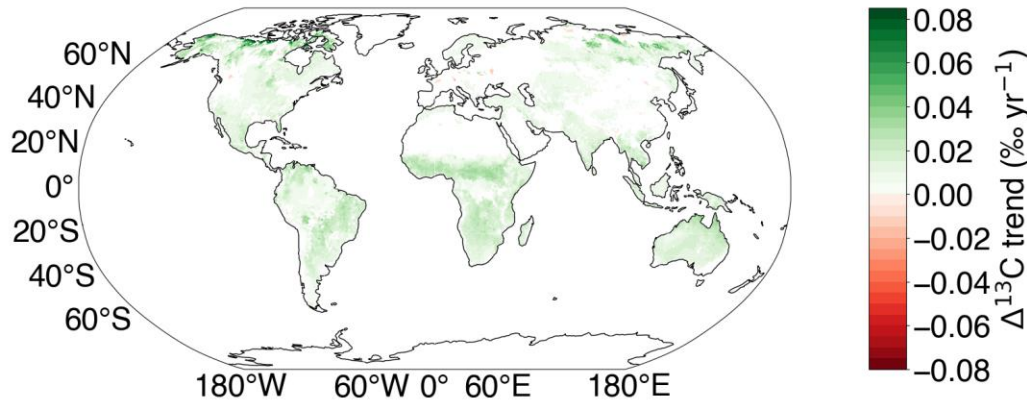

Supplementary Figure 4 Impact of environmental drivers on the fraction of C₄ plants across the globe over 2012–2016. (a) atmospheric CO₂ concentrations (c_a), (b) daytime air temperature (T_{air}), (c) daytime vapour pressure deficit (VPD) and (d) soil moisture (θ).

(a) c_a effect

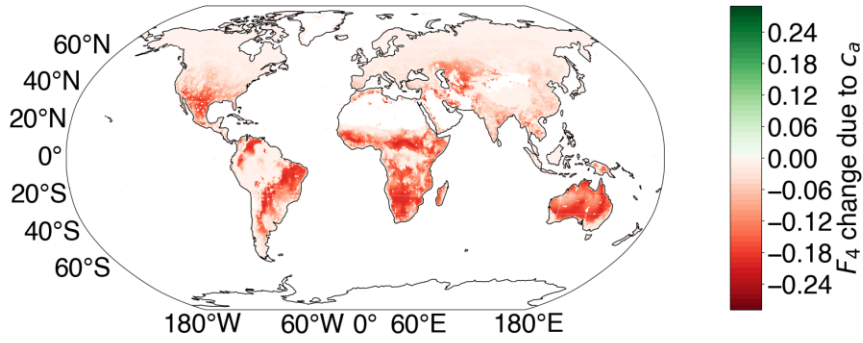

(b) T_{air} effect

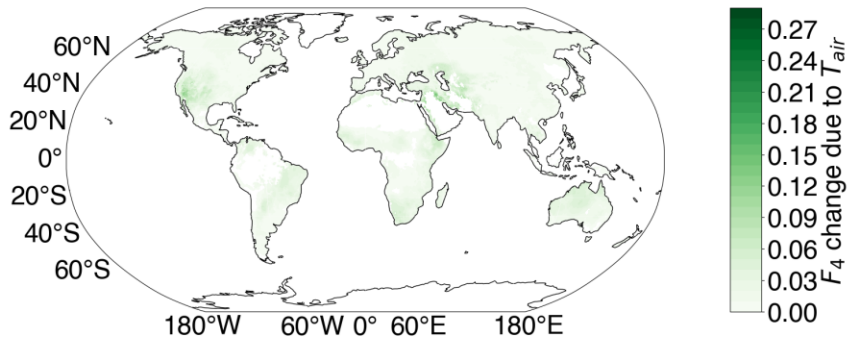

(c) VPD effect

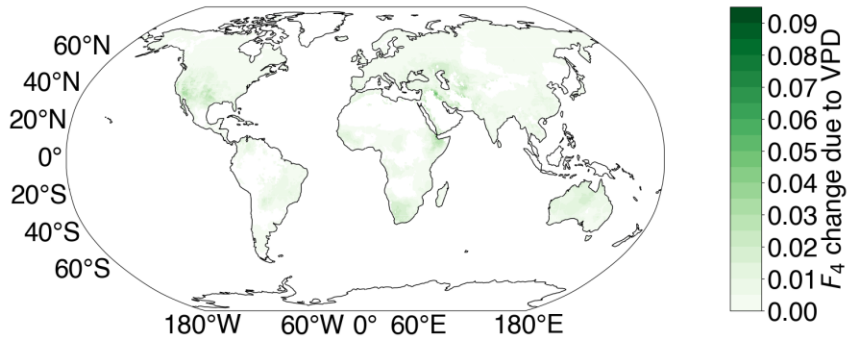

(d) θ effect

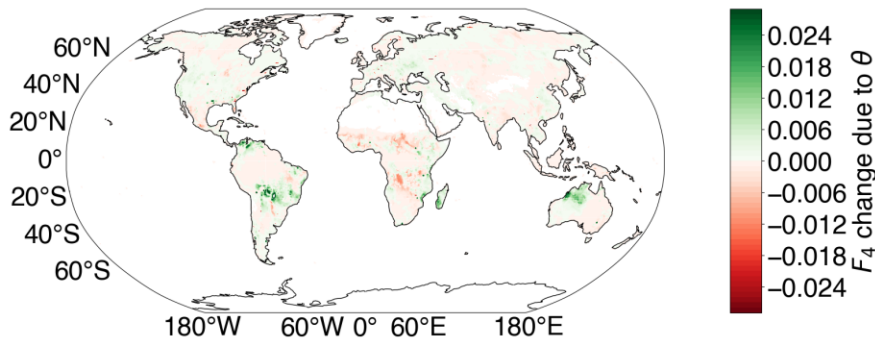

Supplementary Figure 5 Global trends in climate variables. (a) daytime air temperature (T_{air} , $^{\circ}\text{C yr}^{-1}$), (b) daytime vapour pressure deficit (VPD, Pa yr^{-1}) and (c) soil moisture (θ , $\text{m}^3 \text{m}^{-3} \text{yr}^{-1}$) over 1982-2016. Only trends significant at 95% are shown.

(a)

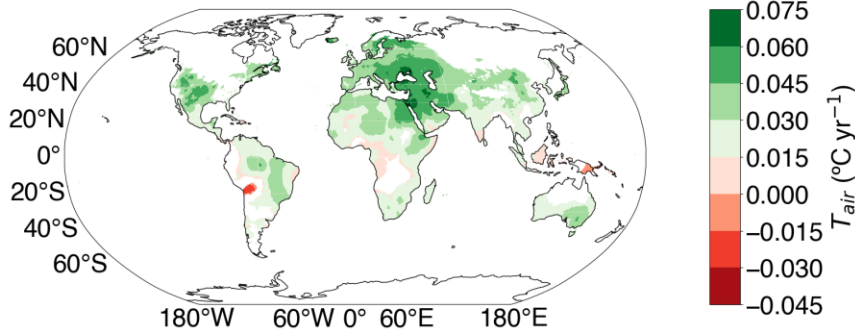

(b)

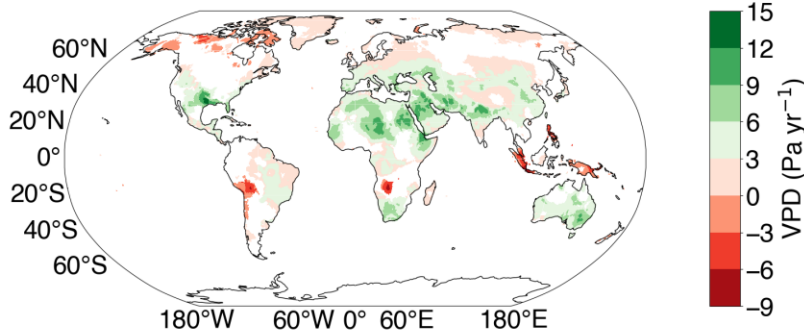

(c)

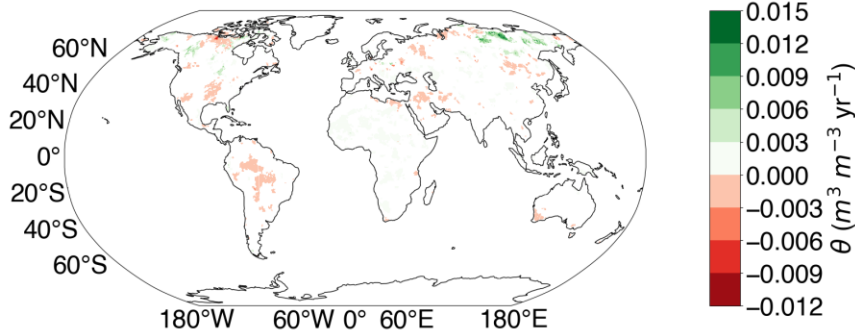

Supplementary Figure 6 Sensitivity of predicted GPP to environmental drivers for C₃ (purple) and C₄ (brown) plants. The standard values are: $T_{\text{air}} = 25^{\circ}\text{C}$, VPD = 0.80 kPa, PPFD = 800 mol m⁻² mth⁻¹, $f\text{APAR} = 0.90$, $c_a = 400 \mu\text{mol mol}^{-1}$, $\theta = 0.4 \text{ m}^3 \text{ m}^{-3}$ and $z = 0 \text{ m}$. The dashed lines in panels (a) and (e) represent GPP sensitivity to T_{air} and c_a when c_a is equal to 300 $\mu\text{mol mol}^{-1}$ and T_{air} is equal to 15°C, respectively.

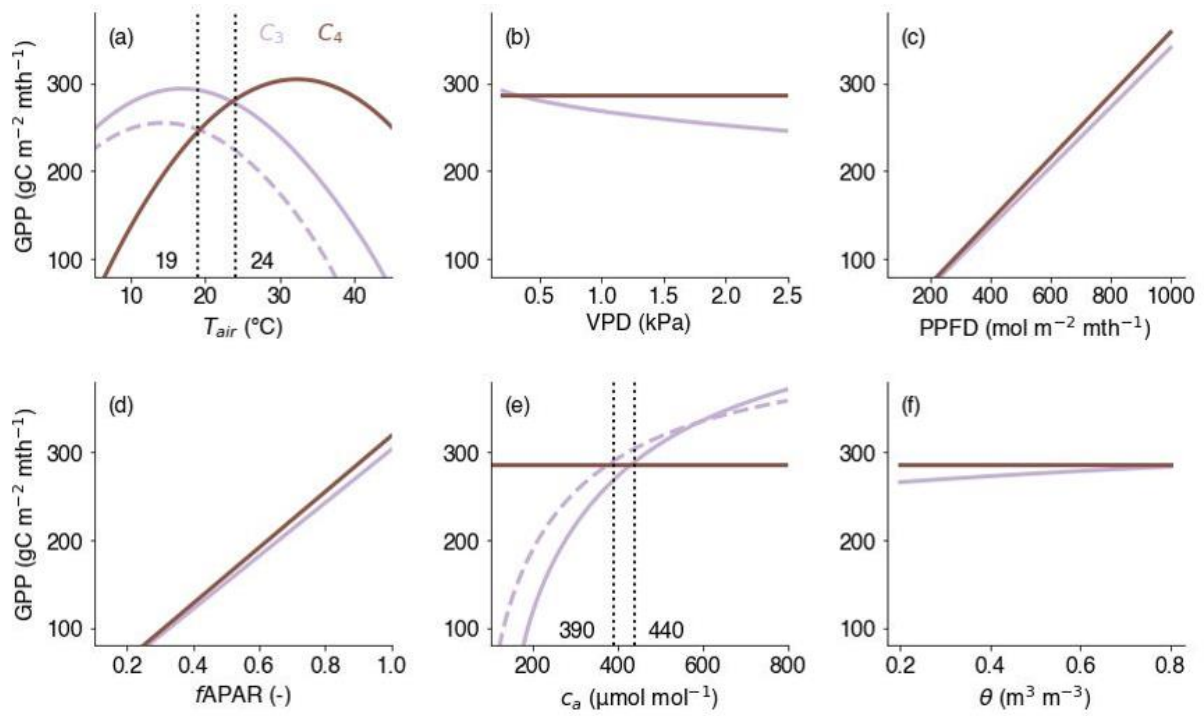

Supplementary Figure 7 Sensitivity of predicted GPP and $\Delta^{13}\text{C}$ to environmental drivers under global environmental conditions over 1982-2016 period. (a, c) C_3 and (b, d) C_4 plants. Standardized coefficients of the multiple linear regressions are shown for each variable.

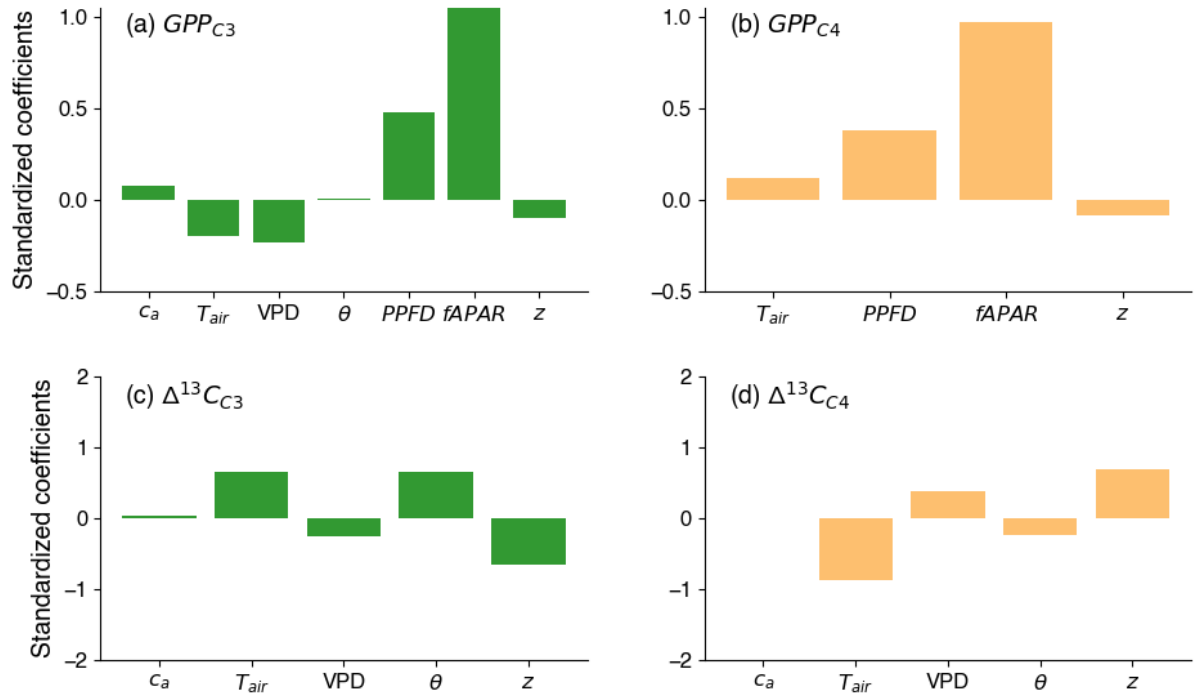

Supplementary Figure 8 Global compilation of leaf and soil isotopic data used to evaluate the model. (a) leaf and (b) soil $\delta^{13}\text{C}$ data. In panel (a) triangles represent sites for C_4 plants while circles are for C_3 plants.

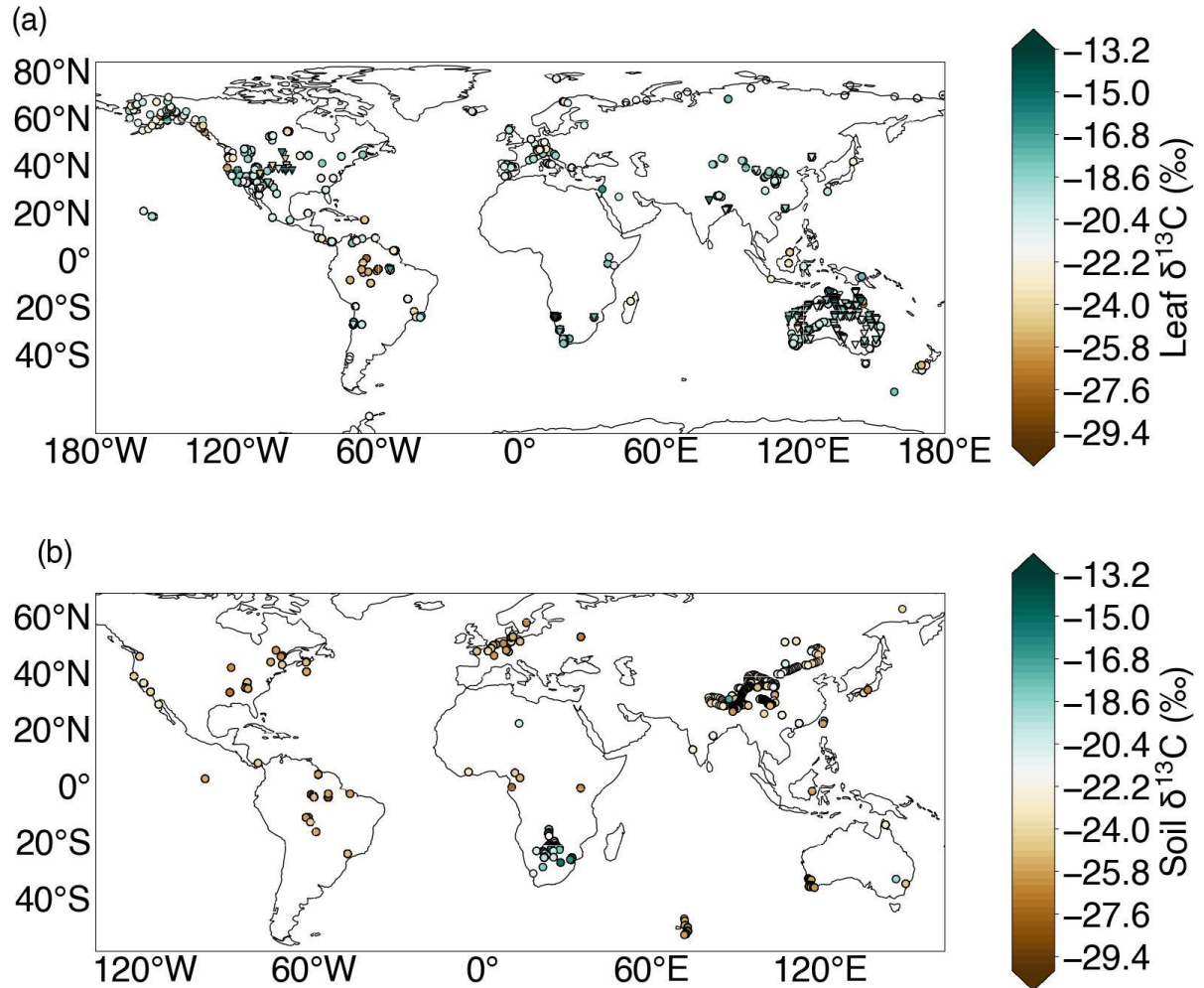

Supplementary Figure 9 Empirical evaluation of the potential influence of plant-available soil water on β values for C₄ plants using 1,161 C₄ leaf gas exchange measurements from Lin et al. (2015)⁶.

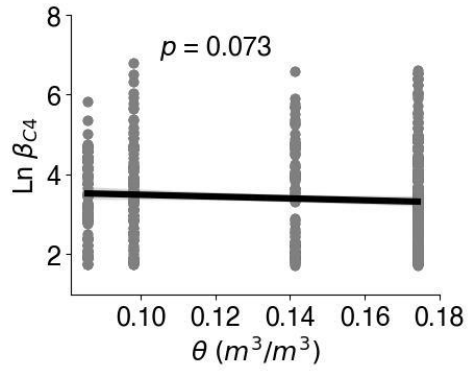

Supplementary Figure 10 Logistic regression of the relationship between the observed contribution of C₄ plants to total ecosystem GPP (Sh_4) derived from [7,8] and the simulated annual C₄ advantage (Adv_4). In light blue is the 95% confidence interval of the regression.

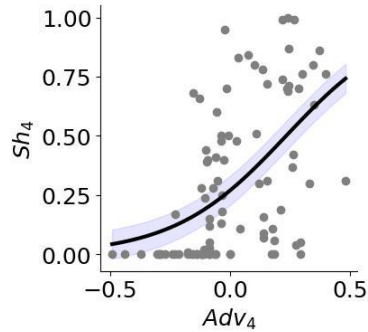

Supplementary Figure 11 Density plots. (a) simulated GPP for C₃ and C₄ plants and (b) percentage of tree cover from the MEaSUREs VCF data set when $Sh_4 > 0.35$. The dotted vertical lines represent the threshold values of 2.8 kgC m⁻² yr⁻¹ when TC is higher than 58%.

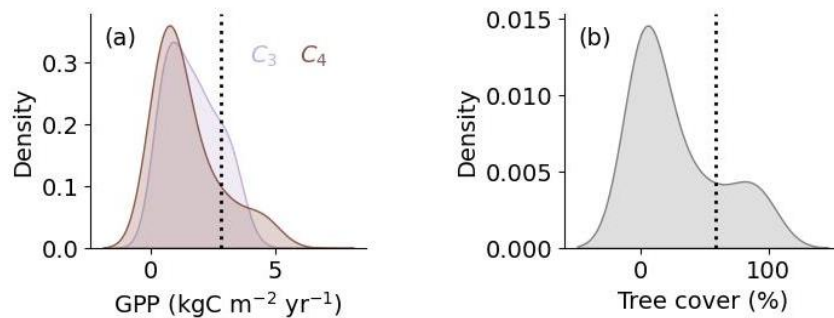

Supplementary Figure 12 Leaf-derived discrimination against ^{13}C (Δ_4) as a function of predicted leaf-internal (c_i) and ambient (c_a) partial pressure of CO_2 (χ). (a) C_3 and (b) C_4 plants.

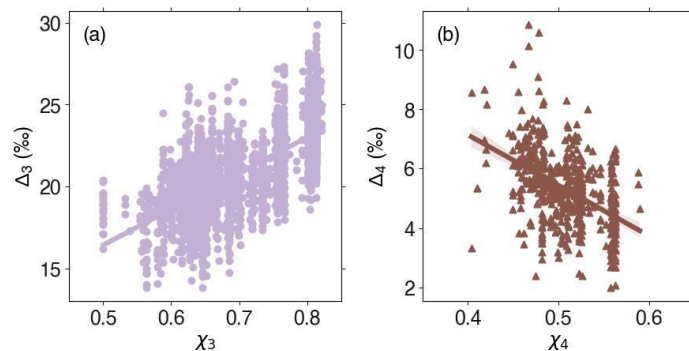

Supplementary References

1. Prentice, I. C., Dong, N., Gleason, S. M., Maire, V. & Wright, I. J. Balancing the costs of carbon gain and water transport: testing a new theoretical framework for plant functional ecology. *Ecol Lett* **17**, 82–91 (2014).
2. Stocker, B. D. *et al.* P-model v1.0: an optimality-based light use efficiency model for simulating ecosystem gross primary production. *Geosci. Model Dev.* **13**, 1545–1581 (2020).
3. Wang, H. *et al.* Towards a universal model for carbon dioxide uptake by plants. *Nat Plants* **3**, 734–741 (2017).
4. Cai, W. & Prentice, I. C. Recent trends in gross primary production and their drivers: analysis and modelling at flux-sites and global scales. *Environ. Res. Lett.* **15**, 124050 (2020).
5. Lavergne, A., Sandoval, D., Hare, V. J., Graven, H. & Prentice, I. C. Impacts of soil water stress on the acclimated stomatal limitation of photosynthesis: insights from stable carbon isotope data. *Glob Chang Biol.* **26**, 7158–7172 (2020).

- 323 6. Lin, Y.-S. *et al.* Optimal stomatal behaviour around the world. *Nat. Clim. Change* **5**, 459–
324 464 (2015).
- 325 7. Paruelo, J. M. & Lauenroth, W. K. Relative Abundance of Plant Functional Types in
326 Grasslands and Shrublands of North America. *Ecol. Appl.* **6**, 1212–1224 (1996).
- 327 8. Paruelo, J. M., Jobbagy, E. G., Sala, O. E., Lauenroth, W. K. & Burke, I. C. Functional
328 and structural convergence of temperate grassland and shrubland ecosystems. *Ecol. Appl.*
329 **8**, 194–206 (1998).
- 330 9. Collatz, G. J., Berry, J. A. & Clark, J. S. Effects of climate and atmospheric CO₂ partial
331 pressure on the global distribution of C₄ grasses: Present, past, and future. *Oecologia* **114**,
332 441–454 (1998).
- 333 10. Sage, R. F., de Melo Peixoto, M., Friesen, P. & Deen, B. C₄ bioenergy crops for cool
334 climates, with special emphasis on perennial C₄ grasses. *J Exp Bot* **66**, 4195–212 (2015).
- 335 11. Luo, X. *et al.* Mapping the global distribution of C₄ vegetation using observations
336 and optimality theory. *Nat. Commun.* **15**, 1219 (2024).
- 337 12. Farquhar, G. D., O’Leary, M. H. & Berry, J. A. On the Relationship between Carbon
338 Isotope Discrimination and the Intercellular Carbon Dioxide Concentration in Leaves.
339 *Aust. J. Plant Physiol.* **9**, 121–137 (1982).
- 340 13. von Caemmerer, S., Ghannoum, O., Pengelly, J. J. & Cousins, A. B. Carbon isotope
341 discrimination as a tool to explore C₄ photosynthesis. *J Exp Bot* **65**, 3459–70 (2014).
- 342 14. Cornwell, W. K. *et al.* Climate and soils together regulate photosynthetic carbon
343 isotope discrimination within C₃ plants worldwide. *Glob. Ecol. Biogeogr.* **27**, 1056–1067
344 (2018).
- 345 15. Graven, H., Keeling, R. F. & Rogelj, J. Changes to Carbon Isotopes in Atmospheric
346 CO₂ Over the Industrial Era and Into the Future. *Glob. Biogeochem. Cycles* **34**, (2020).

16. Meinshausen, M. *et al.* Historical greenhouse gas concentrations for climate modelling (CMIP6). *Geosci. Model Dev.* **10**, 2057–2116 (2017).
17. Köhler, P., Nehrbass-Ahles, C., Schmitt, J., Stocker, T. F. & Fischer, H. A 156 kyr smoothed history of the atmospheric greenhouse gases CO₂, CH₄, and N₂O and their radiative forcing. *Earth Syst. Sci. Data* **9**, 363–387 (2017).
18. Hoesly, R. M. *et al.* Historical (1750–2014) anthropogenic emissions of reactive gases and aerosols from the Community Emissions Data System (CEDS). *Geosci. Model Dev.* **11**, 369–408 (2018).
19. Jones, C. D. *et al.* C4MIP - The Coupled Climate–Carbon Cycle Model Intercomparison Project: experimental protocol for CMIP6. *Geosci. Model Dev.* **9**, 2853–2880 (2016).
20. Andres, R., Boden, T. & Marland, G. Annual Fossil-Fuel CO₂ Emissions: Global Stable Carbon Isotope Signature. Environmental System Science Data Infrastructure for a Virtual Ecosystem; Carbon Dioxide Information Analysis Center (CDIAC), Oak Ridge National Laboratory (ORNL), Oak Ridge, TN (United States) <https://doi.org/10.3334/CDIAC/FFE.DB1013.2017> (2017).
21. Graven, H. *et al.* Compiled records of carbon isotopes in atmospheric CO₂ for historical simulations in CMIP6. *Geosci. Model Dev.* **10**, 4405–4417 (2017).
22. Schubert, B. A. & Jahren, A. H. Global increase in plant carbon isotope fractionation following the Last Glacial Maximum caused by increase in atmospheric pCO₂. *Geology* **43**, 435–438 (2015).
23. Keeling, R. F. *et al.* Atmospheric evidence for a global secular increase in carbon isotopic discrimination of land photosynthesis. *Proc Natl Acad Sci U S A* **114**, 10361–10366 (2017).

- 371 24. Ehleringer, J. R. & Bjorkman, O. Quantum Yields for CO₂ Uptake in C₃ and C₄
372 Plants. *Plant Physiol* **59**, 86–90 (1977).
- 373 25. Skillman, J. B. Quantum yield variation across the three pathways of photosynthesis:
374 not yet out of the dark. *J Exp Bot* **59**, 1647–61 (2008).
- 375 26. Long, S. P. Environmental responses. in *C₄ plant biology* (eds Sage, R. F. & Monson,
376 R. K.) 215–249 (Academic, San Diego, California, US, 1999).
- 377 27. Sage, R. F. & Monson, R. K. *C₄ Plant Biology*. (Academic Press, San Diego, 1999).
- 378 28. Walker, A. P. *et al.* Integrating the evidence for a terrestrial carbon sink caused by
379 increasing atmospheric CO₂. *New Phytol* **229**, 2413–2445 (2021).
- 380 29. Ehleringer, J. R., Cerling, T. E. & Helliker, B. R. C₄ photosynthesis, atmospheric
381 CO₂, and climate. *Oecologia* **112**, 285–299 (1997).
- 382 30. Still, C. J., Berry, J. A., Collatz, G. J. & DeFries, R. S. Global distribution of C₃ and
383 C₄ vegetation: Carbon cycle implications. *Glob. Biogeochem. Cycles* **17**, 6-1-6–14 (2003).
- 384 31. Prentice, I. C. & Harrison, S. P. Ecosystem effects of CO₂ concentration: evidence
385 from past climates. *Clim. Past* **5**, 297–307 (2009).
- 386 32. Wherley, B. G. & Sinclair, T. R. Differential sensitivity of C₃ and C₄ turfgrass
387 species to increasing atmospheric vapor pressure deficit. *Environ. Exp. Bot.* **67**, 372–376
388 (2009).
- 389 33. Ainsworth, E. A. & Long, S. P. 30 years of free-air carbon dioxide enrichment
390 (FACE): What have we learned about future crop productivity and its potential for
391 adaptation? *Glob Chang Biol* **27**, 27–49 (2021).
- 392 34. Sage, R. F., Monson, R. K., Ehleringer, J. R., Adachi, S. & Pearcy, R. W. Some like it
393 hot: the physiological ecology of C₄ plant evolution. *Oecologia* **187**, 941–966 (2018).
- 394 35. Farquhar, G. On the nature of carbon isotope discrimination in C₄ species. *Aust J*
395 *Plant Physiol* **10**, 205–226 (1983).

- 396 36. Dong, N. *et al.* *Global Datasets of Carbon Isotope Composition ($\delta^{13}\text{C}$) for*
397 *Ecological and Earth System Research*. <https://doi.org/10.5281/zenodo.6556096> (2022).
- 398 37. Wang, C. *et al.* Stable isotopic constraints on global soil organic carbon turnover.
399 *Biogeosciences* **15**, 987–995 (2018).
- 400 38. Aranibar, J. N. & Macko, S. A. *SAFARI 2000 Plant and Soil C and N Isotopes,*
401 *Southern Africa, 1995-2000. Data Set.* (2005).
- 402 39. Camino-Serrano, M. *et al.* Including Stable Carbon Isotopes to Evaluate the
403 Dynamics of Soil Carbon in the Land-Surface Model ORCHIDEE. *J Adv Model Earth*
404 *Syst* **11**, 3650–3669 (2019).
- 405 40. Ehleringer, J. R. *et al.* *LBA-ECO CD-02 Carbon, Nitrogen, Oxygen Stable Isotopes in*
406 *Organic Material, Brazil.* (2010).
- 407 41. Lai, Y. *et al.* A data-driven topsoil $\delta^{13}\text{C}$ dataset and the drivers of spatial variability
408 across the Tibetan Plateau. *Earth Syst. Sci. Data Discuss.* [https://doi.org/10.5194/essd-](https://doi.org/10.5194/essd-2021-411)
409 [2021-411](https://doi.org/10.5194/essd-2021-411) (2022) doi:10.5194/essd-2021-411.
- 410 42. Nel, J. A., Craine, J. M. & Cramer, M. D. Correspondence between $\delta^{13}\text{C}$ and $\delta^{15}\text{N}$ in
411 soils suggests coordinated fractionation processes for soil C and N. *Plant Soil* **423**, 257–
412 271 (2018).
- 413 43. Peri, P. L. *et al.* Carbon ($\delta^{13}\text{C}$) and nitrogen ($\delta^{15}\text{N}$) stable isotope composition in
414 plant and soil in Southern Patagonia’s native forests. *Glob. Change Biol.* **18**, 311–321
415 (2012).
- 416 44. Sommer, J. *et al.* Limited carbon and mineral nutrient gain from mycorrhizal fungi by
417 adult Australian orchids. *Am J Bot* **99**, 1133–45 (2012).
- 418 45. Tedersoo, L. *et al.* Enzymatic activities and stable isotope patterns of ectomycorrhizal
419 fungi in relation to phylogeny and exploration types in an afrotropical rain forest. *New*
420 *Phytol* **195**, 832–843 (2012).
